# FOXC1 and FOXC2 regulate growth plate chondrocyte maturation towards hypertrophy in the embryonic mouse limb skeleton

**DOI:** 10.1242/dev.202798

**Published:** 2024-08-22

**Authors:** Asra Almubarak, Qiuwan Zhang, Cheng-Hai Zhang, Noor Abdelwahab, Tsutomu Kume, Andrew B. Lassar, Fred B. Berry

**Affiliations:** ^1^ Department of Medical Genetics, University of Alberta, Edmonton, AB T6G 2E1, Canada; ^2^ Department of Biological Chemistry and Molecular Pharmacology, Blavatnik Institute at Harvard Medical School, 240 Longwood Ave, Boston, MA 02115, USA; ^3^ Department of Surgery, University of Alberta, Edmonton, AB T6G 2E1, Canada; ^4^ Feinberg Cardiovascular and Renal Research Institute, Feinberg School of Medicine, Department of Medicine, Northwestern University, Chicago, IL 60611, USA; ^5^ Women and Children's Health Research Institute, University of Alberta, Edmonton, AB T6G 2E1, Canada

**Keywords:** Endochondral ossification, Bone mineralization, Chondrocyte hypertrophy, Phex

## Abstract

The Forkhead box transcription factors FOXC1 and FOXC2 are expressed in condensing mesenchyme cells at the onset of endochondral ossification. We used the *Prx1-cre* mouse to ablate *Foxc1* and *Foxc2* in limb skeletal progenitor cells. *Prx1-cre;Foxc1*^Δ/Δ^*;Foxc2*^Δ/Δ^ limbs were shorter than controls, with worsening phenotypes in distal structures. Cartilage formation and mineralization was severely disrupted in the paws. The radius and tibia were malformed, whereas the fibula and ulna remained unmineralized. Chondrocyte maturation was delayed, with fewer Indian hedgehog-expressing, prehypertrophic chondrocytes forming and a smaller hypertrophic chondrocyte zone. Later, progression out of chondrocyte hypertrophy was slowed, leading to an accumulation of COLX-expressing hypertrophic chondrocytes and formation of a smaller primary ossification center with fewer osteoblast progenitor cells populating this region. Targeting *Foxc1* and *Foxc2* in hypertrophic chondrocytes with *Col10a1-cre* also resulted in an expanded hypertrophic chondrocyte zone and smaller primary ossification center. Our findings suggest that FOXC1 and FOXC2 direct chondrocyte maturation towards hypertrophic chondrocyte formation. At later stages, FOXC1 and FOXC2 regulate function in hypertrophic chondrocyte remodeling to allow primary ossification center formation and osteoblast recruitment.

## Teaser

**Summary:** Loss of FOXC1 and FOXC2 function in *Prx1*-expressing limb bud mesenchymal progenitors slows chondrocyte differentiation affecting formation of hypertrophic chondrocytes and the primary ossification center.

## INTRODUCTION

In mammals, the limb skeleton, the vertebrae, ribs and parts of the skull are formed through endochondral ossification, in which a cartilaginous template is first formed to grow and shape the bone before being replaced by mineralized tissues (Kozhemyakina et al., 2015). In the limb, mesenchymal progenitors from the lateral plate mesoderm condense and differentiate into chondrocytes, and a subpopulation of these progenitors surrounds this cartilage primordium and forms the perichondrium. Chondrocytes in the bone anlage progress through a series of differentiation events to form a layered, organized structure known as the growth plate (Yeung Tsang et al., 2014; Tickle and Towers, 2020). At the distal ends of the growth plate, immature chondrocytes transition to a highly proliferative state, and stack together to form the columnar zone of the growth plate. These columnar chondrocytes then exit from the cell cycle to become prehypertrophic chondrocytes (PHCs) that express Indian hedgehog (*Ihh*), which regulates proliferation and differentiation of chondrocytes and osteoblast formation (Karp et al., 2000; Vortkamp et al., 1996). PHCs enlarge in volume and become hypertrophic chondrocytes (HCs) that form the interface between bone and cartilage and regulate the subsequent ossification process. HCs express matrix metalloproteinase (MMP) 13, which degrades the chondrocyte extracellular matrix (ECM) and, along with chondroclasts and osteoclasts, helps in the formation of the primary ossification center (POC). HCs also produce vascular endothelial growth factor (VEGF), which promotes the invasion of blood vessels that populate the POC (Gerber et al., 1999; Zelzer et al., 2004). HCs are then either removed or differentiate to form some of the osteoblasts in the POC (Yang et al., 2014). Osteoblast progenitors migrate from the surrounding perichondrium/periosteum along with blood vessels to mineralize the POC (Maes et al., 2010).

The forkhead box C1 and C2 (FOXC1 and FOXC2) transcription factors are important regulators of skeletal development (Almubarak et al., 2021; Kume et al., 1998; Winnier et al., 1997; Yoshida et al., 2015). Abundant expressions of FOXC1 and FOXC2 are observed in the condensing skeletal mesenchyme of the limb and the vertebrae in mice by embryonic day (E) 11.5 (Almubarak et al., 2021; Hiemisch et al., 1998b). Later, as chondrocytes become more differentiated, expression of both FOXC1 and FOXC2 is reduced in the central bone anlages and enriched in the surrounding perichondrium (Almubarak et al., 2021; Hiemisch et al., 1998b). Although the expression of FOXC1 and FOXC2 broadly overlaps during endochondral development, distinct expression patterns are observed. For example, FOXC1 expression is elevated at the proximal and distal limb anlage, whereas FOXC2 expression is abundant in central regions of this structure (Almubarak et al., 2021; Hiemisch et al., 1998b). *FOXC1* mutations in humans cause Axenfeld Rieger syndrome, an autosomal dominant condition affecting the eyes and craniofacial skeleton (Mears et al., 1998). It is thought that haploinsufficiency from inactivating a single *FOXC1* allele is an underlying cause of this disorder, although patients with *FOXC1* gene duplications can also present with Axenfeld Rieger anomalies (Lehmann et al., 2000). Moreover, individuals with single chromosomal microdeletions of *FOXC1* present with numerous skeletal anomalies, including epiphyseal dysplasia of the humeral and femoral heads, and slender long bones (Garza Flores et al., 2023; Kannu et al., 2006). *FOXC2* mutations causes Lymphodema-Distichiasis syndrome, which can present with craniofacial and vertebral malformations (Brice et al., 2002). *Foxc1^−/−^* or *Foxc2^−/−^* mice (germline mutations) display an absence of mineralization in the skull, vertebral column and rib cage, whereas the bones in the limbs or appendicular skeleton are reduced in length but are mineralized (Hong et al., 1999; Kume et al., 1998; Winnier et al., 1997). As FOXC1 and FOXC2 share a near identical DNA-binding domain, with only two conservative amino acid substitutions in both mouse and human genes and over 75% sequence conservation in the transcriptional activation domains (Hiemisch et al., 1998a; Pierrou et al., 1994; Sasaki and Hogan, 1993), it is likely that FOXC1 and FOXC2 may compensate for the loss of the other. Compound *Foxc1^−/−^;Foxc2^−/−^* mice die at ∼E9 due to failure in cardiovascular development, and before any skeletal structures are formed, preventing any analysis of the possible association between the two transcription factors in endochondral ossification to be studied (Kume et al., 2001).

To address potential compensation between *Foxc1* and *Foxc2* during skeletal development, we previously created a conditional mouse model that deleted both genes in the chondrocyte lineage (Almubarak et al., 2021). These *Col2a1-cre;Foxc1*^Δ/Δ^*;Foxc2*^Δ/Δ^ mutants displayed impaired chondrocyte differentiation in embryogenesis and led to a general skeletal hypoplasia that affected the axial skeleton more so than the bones in the limb. The cervical vertebrae were absent in the *Col2a1-cre;Foxc1*^Δ/Δ^*;Foxc2*^Δ/Δ^ mutants and the thoracic and lumbar vertebral bodies failed to differentiate past chondrocyte condensations and little mineralization was detected. In the limb, the long bones were bowed, displayed delayed chondrocyte maturation and a reduction in mineralization length. Given the abundant expression of *Foxc1* and *Foxc2* in condensing limb bud mesenchyme (Almubarak et al., 2021; Hiemisch et al., 1998b), such phenotypic differences between the axial and appendicular skeleton were surprising. The mesenchyme that forms the axial skeleton is derived from the sclerotome. As the *Col2a1-cre* transgene is active in sclerotome cells before the onset of chondrogenesis, but only becomes active in the limb once condensations form (Ovchinnikov et al., 2000), we thought that this earlier timing of the deletion of *Foxc1* and *Foxc2* in axial elements might explain the phenotypic differences we observe in the axial versus appendicular skeleton. To address these issues, we deleted *Foxc1* and *Foxc2* in the limb at an earlier developmental stage than when *Col2-cre* is active using *Sox9-cre* and in *Prx1-cre* mice.

## RESULTS

### Impaired formation of cartilaginous elements by early deletion of *Foxc1* and *Foxc2* in *Sox9-cre*-expressing cells

We examined chondrocyte differentiation in embryos when *Foxc1* and *Foxc2* were targeted in *Sox9*-expressing cells (*Sox9^ires-Cre^*; Akiyama et al., 2005; Sasman et al., 2012). Embryos at E12.5 that lacked the *Cre* driver displayed Alcian Blue staining of both their paraxial mesoderm-derived and appendicular skeletal structures. In contrast, *Sox9^ires-Cre/+^;Foxc1*^Δ/Δ^*;Foxc2*^Δ/Δ^ littermates displayed a dramatic loss of Alcian Blue staining in their paraxial mesoderm, but maintained that in their developing limb buds (Fig. S1). *Sox9^ires-Cre/+^;Foxc1*^Δ/Δ^*;Foxc2^+^*^/Δ^ and *Sox9^ires-Cre/+^;Foxc1^+^*^/Δ^*;Foxc2*^Δ/Δ^ embryos displayed intermediate levels of Alcian Blue staining in their paraxial mesoderm. These results indicate that FOXC1 and FOXC2 have overlapping roles in promoting chondrogenesis of the paraxial-derived mesoderm. In addition, it suggests that other factors may work with FOXC1 and FOXC2 to promote the initiation of chondrogenesis in the appendicular skeleton.

**Fig. 1.**
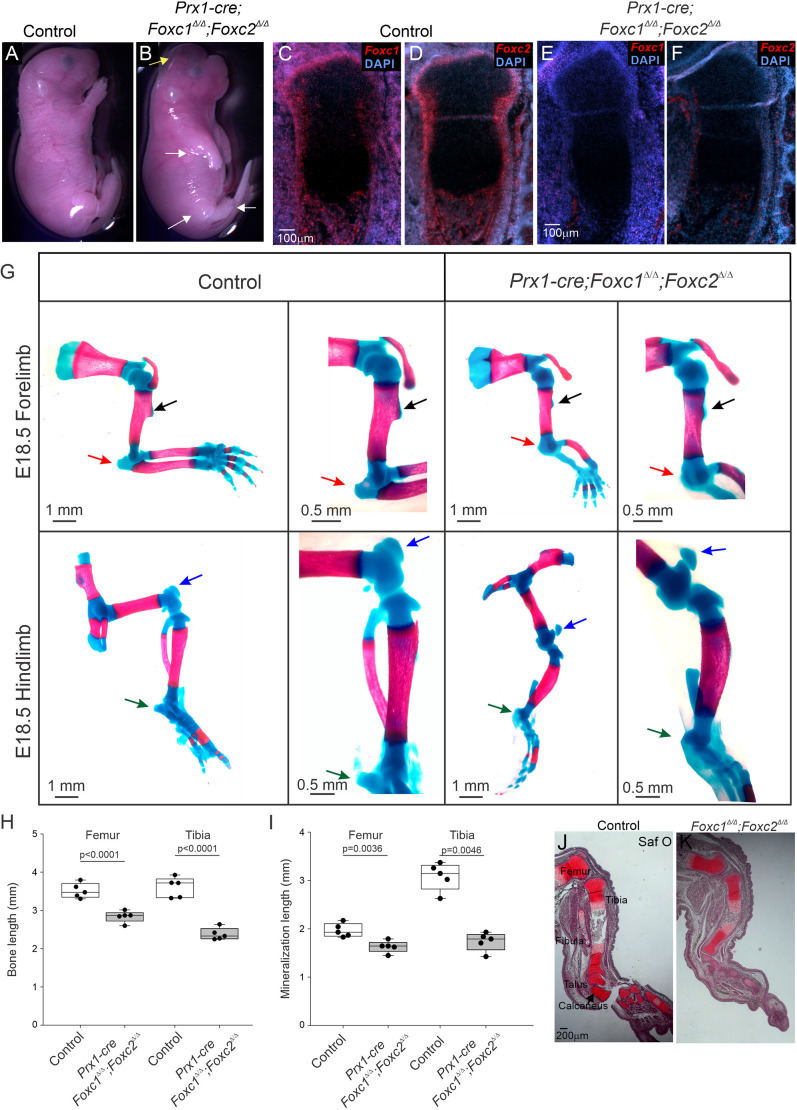
**FOXC1 and FOXC2 play crucial roles in the formation of the distal regions of the appendicular skeleton and support the growth of the bone eminences.** (A,B) E18.5 control (A) and *Prx1-cre;Foxc1*^Δ/Δ^*;Foxc2*^Δ/Δ^ (B) embryos. The *Prx1-cre* mutant mice show the development of exencephaly (yellow arrow) and a reduction in forelimb and hindlimb size (white arrows). (C-F) *In situ* hybridization detection of *Foxc1* and *Foxc2* mRNA expression in control (C,D) and *Prx1-cre;Foxc1*^Δ/Δ^*;Foxc2*^Δ/Δ^ (E,F) embryos at E16.5 confirms *Foxc1* and *Foxc2* deletion in the limb. (G) Whole skeletal staining of the appendicular skeleton in control and *Prx1-cre;Foxc1*^Δ/Δ^*;Foxc2*^Δ/Δ^ embryos at E18.5. Arrows indicate the deltoid tuberosity (black arrows) and olecranon (red arrows) in the forelimb, and the calcaneal tuberosity (green arrows) and the patella (blue arrows) in the hindlimb. (H,I) Total bone length (H) and mineralization length (I) was measured in the femurs and tibias of control and *Prx1-cre;Foxc1*^Δ/Δ^*;Foxc2*^Δ/Δ^ embryos at E18.5 from five littermate pairs. Statistical analysis was performed by unpaired two-tailed Student's *t*-test. Box plots show the median value and the 25th and 75th percentile. Whiskers indicate the 10th and 90th percentile. Dots represent individual data points. (J,K) Safranin O staining of control and *Prx1-cre;Foxc1*^Δ/Δ^*;Foxc2*^Δ/Δ^ hindlimb at E16.5.

*Sox9^ires-Cre/+^;Foxc1*^Δ/Δ^*;Foxc2*^Δ/Δ^ mice embryos were not viable after E12.5, preventing further analysis of skeletal development. We then used *Prx1-cre* to delete these genes in limb bud mesenchyme which targets both chondrocyte and osteoblast progenitors (Logan et al., 2002). *Prx1-cre;Foxc1*^Δ/Δ^*;Foxc2*^Δ/Δ^ embryos died shortly after birth and exhibited smaller limbs and paws with abnormal forelimb positioning that resembled decerebrate posture (Fig. 1A,B, white arrow) and exencephaly (yellow arrow). Expression of both *Foxc1* and *Foxc2* mRNA at E16.5 was observed in the skeletal structures of control littermates, but not detected in limb skeletal structures of *Prx1-cre;Foxc1*^Δ/Δ^*;Foxc2*^Δ/Δ^ embryos (Fig. 1C-F), confirming the successful deletion of both genes. Loss of *Foxc1* and *Foxc2* in the developing limb bud drastically affected zeugopod and autopod formation, although the overall bone and mineralization length of all bones including the stylopod (femur) were reduced (Fig. 1G-I). The *Prx1-cre* mutant limbs showed a severe stunting of skeletal elements in the zeugopod and a thinning or loss of autopod cartilage elements. Cartilage formation (Safranin O staining) appeared to be reduced in the hindlimb autopod of *Prx1-cre;Foxc1*^Δ/Δ^*;Foxc2*^Δ/Δ^ embryos compared with controls at E16.5 (Fig. 1J,K). In both the fore- and hindlimbs of *Prx1-cre;Foxc1*^Δ/Δ^*;Foxc2*^Δ/Δ^ embryos, many tendon-bone attachment elements were smaller than in control littermates. The deltoid tuberosity, the olecranon in the forelimb, and the calcaneal tuberosity and the patella in the hindlimb, did not significantly grow in *Prx1-cre;Foxc1*^Δ/Δ^*;Foxc2*^Δ/Δ^ embryos (Fig. 1G). These findings signify the importance of *Foxc1* and *Foxc2* in both the proper formation of the limb skeleton cartilage elements and the subsequent development of the bone eminences in the fore- and hindlimbs.

### FOXC1 and FOXC2 regulation of endochondral ossification varies temporally during endochondral bone development

**Fig. 2.**
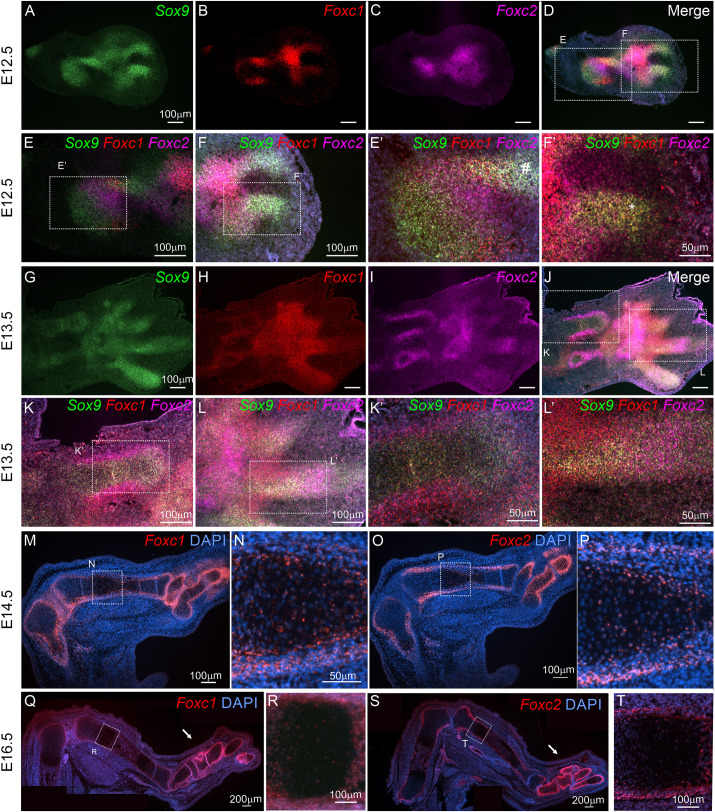
**Dynamic expression of *Foxc1* and *Foxc2* mRNA in the developing hindlimb.** (A-T) Hindlimb spatial and temporal expression patterns of *Foxc1* and *Foxc2* mRNAs were determined at E12.5-E16.5 in the developing hindlimb. Multiplex *in situ* hybridization compared location of the *Sox9*, *Foxc1* and *Foxc2* mRNAs at E12.5 (A-F) and at E13.5 (G-L) in the developing autopod and zeugopod elements. *Foxc1* and *Foxc2* expression in the hindlimb at E14.5 (M-P) and E16.5 (Q-T). Images presented are representative of results from three embryos analyzed per time point.

Loss of *Foxc1* and *Foxc2* in the developing limb bud affected the distal skeletal elements (zeugopod; autopod) more severely than the proximal parts (stylopod). We sought to determine whether spatial expression of *Foxc1* and *Foxc2* could account for the differences in severity in distal versus proximal skeletal elements in *Prx1-cre;Foxc1*^Δ/Δ^*;Foxc2*^Δ/Δ^ embryos. We first compared expression patterns of *Foxc1* and *Foxc2*, along with *Sox9*, in the developing hindlimb at E12.5 and E13.5 (Fig. 2A-L′). At E12.5, chondrogenic condensations were detected by *Sox9* expression in the limb (Fig. 2A). In the E12.5 autopod digit anlage, *Foxc1* mRNA overlapped with *Sox9* expression; however, *Foxc2* was not detected in the digit but was in proximal autopod regions (Fig. 2A-D,F,F′, asterisk). Distinct expression patterns emerged in the zeugopod regions where areas of the highest expression for either *Sox9*, *Foxc1* and *Foxc2* did not appear to overlap with the highest expression region for the other genes (Fig. 2E,E′), although a smaller region in the anterior limb bud contained strong *Sox9* and *Foxc1* signal expression (Fig. 2E,E′, hashtag). At E13.5, we observed continued overlapping *Sox9* and *Foxc1* mRNA expression in the digits, with *Foxc2* mRNA signal emerging in this region (Fig. 2G-J,L,L′). In the zeugopod, *Sox9* expression demarcates the chondrocyte of the newly forming tibia and fibula, and strong *Foxc1* and *Foxc2* mRNA signal surrounds this *Sox9*-expressing region, although lower levels of *Foxc1* and *Foxc2* mRNA were detected along with *Sox9* in the chondrocyte regions (Fig. 2G-J,K,K′). By E14.5, strong *Foxc1* and *Foxc2* mRNA signals were detected in the perichondrium in the developing tarsals and metatarsals, with less intense signal detected in chondrocyte regions of these structures (Fig. 2M,O). In the tibia, *Foxc1* and *Foxc2* expression is also prominent in the perichondrium of epiphysis compared with a lower signal detected in the growth plate and HCs as well as the perichondrium surrounding the metaphysis (Fig. 2N,P). *Foxc1* mRNA was also abundant in the interzone region between the femur and tibia, whereas *Foxc2* expression was absent or reduced in this region along with the distal portions of the tibia epiphysis. By E16.5, *Foxc1* and *Foxc2* mRNA levels become further restricted to the tibia perichondrium, with reduced expression levels detected in HC (Fig 2Q-T). Thus, the expression of *Foxc1* and *Foxc2* is most intense in less mature skeletal elements and becomes restricted to the perichondrium later in development, with reduced comparable expression in the growth plate chondrocytes.

### Loss of *Foxc1* and *Foxc2* reduced proliferation during early growth plate development

**Fig. 3.**
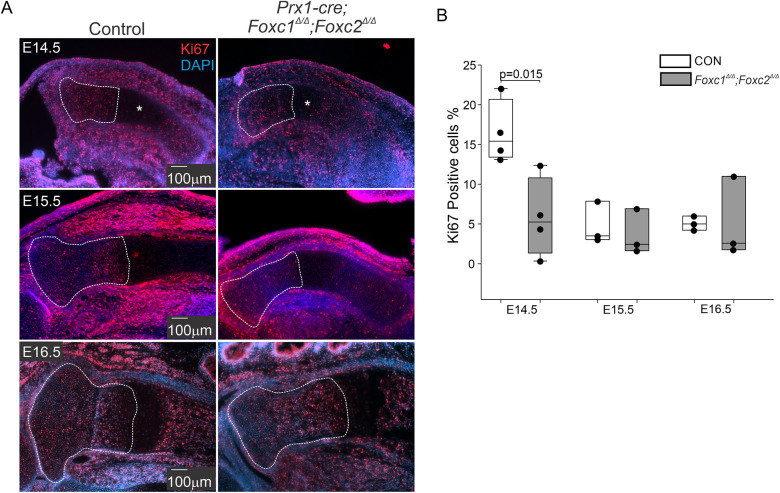
**Cell proliferation is reduced in *Prx1-cre;Foxc1*^Δ/Δ^*;Foxc2*^Δ/Δ^ limbs at E14.5.** (A) Cell proliferation in the proximal tibia growth plate was assessed by KI67 IF. Representative micrographs for control and *Prx1-cre;Foxc1*^Δ/Δ^*;Foxc2*^Δ/Δ^ proximal tibia sections from E14.5, E15.5 and E16.5 are shown. (B) The percentage of KI67-positive cells was determined in the outlined area from control and *Prx1-cre;Foxc1*^Δ/Δ^*;Foxc2*^Δ/Δ^ proximal tibia growth plates at E14.5, E15.5 and E16.5. The HC zone at E14.5 is indicated by an asterisk. Data presented are from three (E15.5 and E16.5) or four (E14.5) embryos per genotype at each age. Statistical analysis was performed by one-way ANOVA. Box plots show the median value and the 25th and 75th percentile. Whiskers indicate the 10th and 90th percentile. Dots represent individual data points.

We tested whether reduced proliferation accounted for the reduced size of the mutant limbs by measuring KI67 immunofluorescence (IF) (Gerdes et al., 1984) in the growth plates of control and *Prx1-cre;Foxc1*^Δ/Δ^*;Foxc2*^Δ/Δ^ embryonic tibias. Control limbs revealed the highest number of KI67-positive cells in the proximal tibia growth plate at E14.5 in comparison with later time points (Fig. 3). The tibia growth plate in E14.5 *Prx1-cre;Foxc1*^Δ/Δ^*;Foxc2*^Δ/Δ^ embryos exhibited an ∼50% reduction in the percentage of KI67-positive cells compared with the control limbs (Fig. 3). Of note, the length of the tibia is noticeably smaller particularly, the HC zone (asterisk). No changes in proliferation activity were detected between control and mutant limbs at E15.5 and E16.5. These results suggest that FOXC1 and FOXC2 act during a narrow window (before E15.5) to regulate chondrocyte proliferation.

**Fig. 4.**
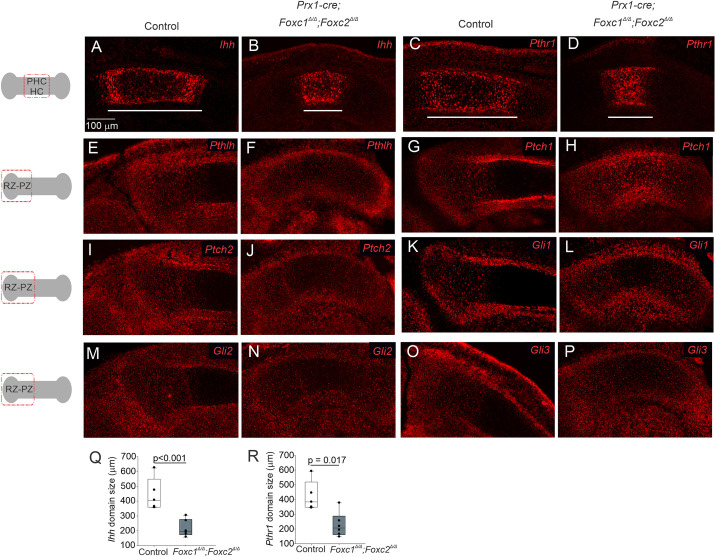
***Ihh* expression is reduced in *Prx1-cre;Foxc1*^Δ/Δ^*;Foxc2*^Δ/Δ^ prehypertrophic chondrocytes at E14.5.** (A-P) Assessment of the IHH-PTHLH signaling axis function in *Prx1-cre;Foxc1*^Δ/Δ^*;Foxc2*^Δ/Δ^ embryos. The position in the growth plate for micrographs is indicated in the schematic on the left. Expression of *Ihh* (A,B), *Pth1r* (C,D), *Pthlh* (E,F), *Ptch1* (G,H), *Ptch2* (I,J),*Gli1* (K,L), *Gli2* (M,N) and *Gli3* (O,P) was assessed by *in situ* hybridization in the tibia of control and *Prx1-cre;Foxc1*^Δ/Δ^*;Foxc2*^Δ/Δ^ embryos at E14.5. (Q,R) The length of *Ihh* (Q) and *Pthr1* (R) -expressing regions (white bars, A-D) was measured in control and *Prx1-cre;Foxc1*^Δ/Δ^*;Foxc2*^Δ/Δ^ embryos. HC, hypertrophic chondrocytes; PHC, prehypertrophic chondrocytes; PZ, proliferating zone; RZ, resting zone. Data are representative of five littermate pairs. Statistical analysis performed using unpaired two-tailed Student's *t*-test. Box plots show the median value and the 25th and 75th percentile. Whiskers indicate the 10th and 90th percentile. Dots represent individual data points.

It has been reported that Foxc1 regulates IHH-GLI signaling to control endochondral ossification (Yoshida et al., 2015). We investigated whether expression of IHH and PTHLH signaling components that regulate chondrocyte proliferation were affected in the growth plate of *Prx1-cre;Foxc1*^Δ/Δ^*;Foxc2*^Δ/Δ^ embryos. The size of the *Ihh*- and parathyroid-like hormone receptor (*Pth1r*)-expressing regions forming the PHCs were smaller in the *Prx1-cre;Foxc1*^Δ/Δ^*;Foxc2*^Δ/Δ^ embryos (Fig. 4A-D;Q,R). Their expression was confined to a smaller domain in the center of the newly forming HC zone compared with the distally displaced signal in the control littermates. *Pthlh* mRNA localized to the perichondrium and resting zone (RZ) in control and *Prx1-cre;Foxc1*^Δ/Δ^*;Foxc2*^Δ/Δ^ embryos; however, expression extend into the newly forming HZ in the mutant embryos (Fig. 4E,F). *Ptch1* and *Ptch2* encode receptors for IHH, and their expression is induced in response to ligand binding; thus, their expression can be used to monitor active hedgehog signaling (Alman, 2015). *Ptch1* and *Ptch2* mRNA were detected in both the proliferating zone (PZ) chondrocytes and in the perichondrium that surrounded *Ihh-*expressing cells in the tibia of E14.5 control embryos (Fig. 4G-J). In the *Prx1-cre;Foxc1*^Δ/Δ^*;Foxc2*^Δ/Δ^ embryos, *Ptch1* and *Ptch2* mRNA expression were localized in a similar pattern that surrounded the smaller *Ihh-*expressing region. In the control embryos, *Gli1*, *Gli2* and *Gli3* mRNAs were detected in the RZ and PZ chondrocytes as well as the perichondrium (Fig. 4K,M,O); these transcripts were detected in similar regions in the *Prx1-cre;Foxc1*^Δ/Δ^*;Foxc2*^Δ/Δ^ mutants (Fig. 4L,N,P). Collectively, these data indicate that the absence of *Foxc1* and *Foxc2* reduces the size of the *Ihh-*expressing PHC zone. Further, although the signaling components needed to mediate IHH-PTHLH signaling are functioning in *Prx1-cre;Foxc1*^Δ/Δ^*;Foxc2*^Δ/Δ^ embryos their spatial distribution was altered in these mutants at this time point.

To determine whether reduced *Ihh* expression and chondrocyte maturation was affected at earlier time points in *Prx1-cre;Foxc1*^Δ/Δ^*;Foxc2*^Δ/Δ^ embryos, we isolated RNA from hindlimb zeugopod elements at E13.5 for qRT-PCR analysis. *Foxc2* mRNA levels were all but undetectable in *Prx1-cre;Foxc1*^Δ/Δ^*;Foxc2*^Δ/Δ^ embryos and the level of *Foxc1* mRNA in the mutants was 25% that of controls, indicating that excision of *Foxc1* by *Prx1-cre* may not be complete at E13.5 and may explain some phenotypic differences in the proximal and distal limb skeleton as deletion in the proximal elements may be delayed, although this expression may reflect *Foxc1* expressed in tissues not targeted by *Prx1-cre*. We also observed a reduction in *Ihh* mRNA levels (1.5× lower) and *Col10a1* (*ColX*) mRNA levels (60× lower) in the hindlimbs of *Prx1-cre;Foxc1*^Δ/Δ^*;Foxc2*^Δ/Δ^ embryos compared with controls, whereas expression of *Col2a1*, *Pthlh* and *Fgfr3* was unchanged (Fig. S2A). We examined COLX protein localization in the limbs of E13.5 embryos. We were unable to detect COLX protein accumulation in the hindlimbs of control or mutant embryos at this stage; however, we did detect COLX protein in the newly forming hypertrophic zone of the humerus of E13.5 control embryos but not in *Prx1-cre;Foxc1*^Δ/Δ^*;Foxc2*^Δ/Δ^ mutants (Fig. S2B).

### Alterations in HC zone growth in *Prx1-cre;Foxc1*^Δ/Δ^*;Foxc2*^Δ/Δ^ embryos

**Fig. 5.**
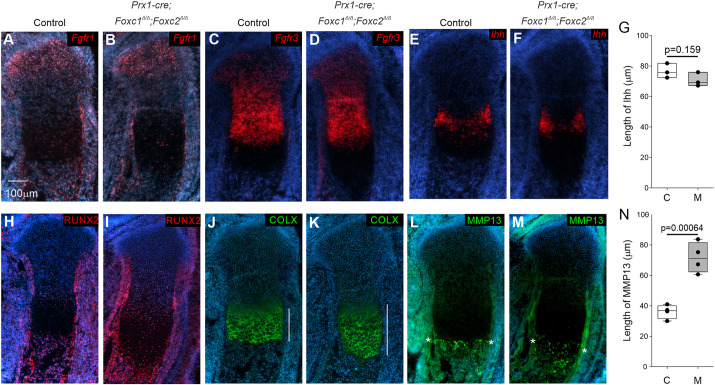
**Expanded hypertrophic chondrocyte zone in growth plate of E16.5 *Prx1-cre;Foxc1*^Δ/Δ^*;Foxc2*^Δ/Δ^ embryos.** (A-N) Chondrocyte differentiation and growth plate organization was examined in proximal tibias at E16.5 from control and *Prx1-cre;Foxc1*^Δ/Δ^*;Foxc2*^Δ/Δ^ embryos using *in situ* hybridization or immunofluorescence. *Fgfr1* (resting zone) (A,B), *Fgfr3* (columnar chondrocytes) (C,D), Ihh (prehypertrophic chondrocytes) (E-G), RUNX2 (prehypertrophic chondrocytes) (H,I), COLX protein (hypertrophic chondrocytes) (J,K) and MMP13 (late hypertrophic chondrocytes) (L-N). White lines in J and K indicate the length of COLX-expressing cells. Asterisks in L and M indicate MMP13-positive HC chondrocytes. For graphs in G and N, control embryos are denoted with C and *Prx1-cre;Foxc1*^Δ/Δ^*;Foxc2*^Δ/Δ^ embryos with M. Data are representative of a minimum of three littermate pairs. Statistical analyses were performed using unpaired two-tailed Student's *t*-test. Box plots show the median value and the 25th and 75th percentile. Whiskers indicate the 10th and 90th percentile. Dots represent individual data points.

We next evaluated chondrogenic differentiation and growth plate organization in *Prx1-cre;Foxc1*^Δ/Δ^*;Foxc2*^Δ/Δ^ embryos at E16.5. No anomalies were detected in the formation of the RZ, PZ and PHC zone of the tibia growth plates in E16.5 mutant embryos (Fig. 5). We found that *Fgfr1* and *Fgfr3* mRNAs were detected in similar patterns in the RZ and PZ chondrocytes, respectively, in both E16.5 control and mutant growth plates (Fig. 5A-D). In addition, both *Ihh* mRNA and RUNX2 protein were similarly localized to the PHC zone in both control and mutant limbs at E16.5 (Fig. 5E-I). We observed an expanded COLX IF signal in the HC zone in the absence of *Foxc1* and *Foxc2* (Fig. 5J,K). MMP13 protein localized to the terminal differentiated HC zone was expanded by nearly two times in the *Prx1-cre;Foxc1*^Δ/Δ^*;Foxc2*^Δ/Δ^ embryos compared with controls (Fig. 5L-N). Taken together, our findings suggest that the initial delay in chondrocyte maturation (reduced size of Ihh-expressing cells) at E14.5 is normalized at later stages of development, and that cells producing COLX and MMP13 proteins were persistent in growth plates of *Prx1-cre;Foxc1*^Δ/Δ^*;Foxc2*^Δ/Δ^ embryos, likely from impaired HC remodeling.

### Regulation of chondrocyte hypertrophy is altered in *Prx1-cre;Foxc1*^Δ/Δ^*;Foxc2*^Δ/Δ^ mice

**Fig. 6.**
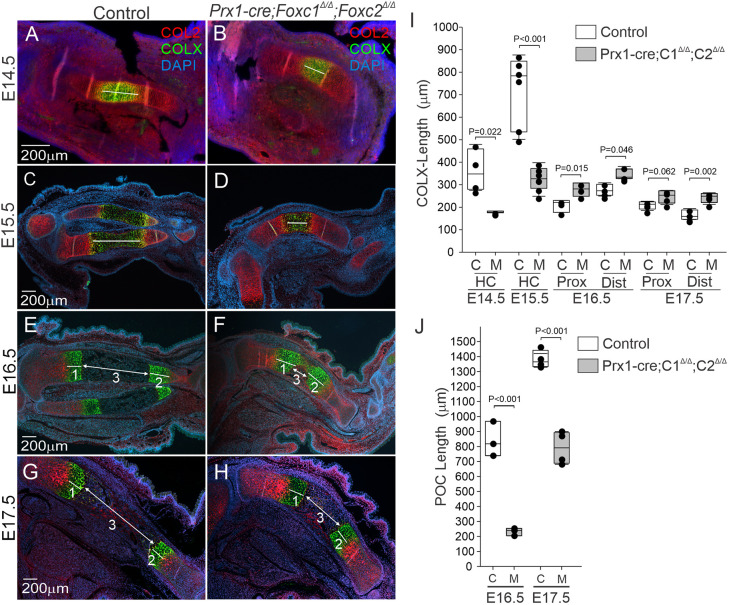
**Progression through chondrocyte hypertrophy and formation of the primary ossification center was delayed in *Prx1-cre;Foxc1*^Δ/Δ^*;Foxc2*^Δ/Δ^ embryos.** (A-H) Formation and growth of the hypertrophic chondrocyte zone was analyzed by COLX IF (green) at E14.5 (A,B), E15.5 (C,D), E16.5 (E,F) and E17.5 (G,H). (I) Length of COLX signal from control (C) or *Prx1-cre;Foxc1*^Δ/Δ^*;Foxc2*^Δ/Δ^ (M) embryos is indicated by the white line. The proximal (Prox) and distal (Dist) COLX-expressing zones were measured in E16.5 and E17.5 embryos. (J) The size of the POC was determined by measuring the distance between terminal COLX expression zones at E16.5 and E17.5. (1) COLX-expressing hypertrophic chondrocytes proximal domain (1-HC-PD); (2) COLX-expressing hypertrophic chondrocyte distal domain (2-HC-DD); (3) Primary ossification center. Statistical analysis was performed using unpaired two-tailed Student's *t*-test. Box plots show the median value and the 25th and 75th percentile. Whiskers indicate the 10th and 90th percentile. Dots represent individual data points. Data were obtained from the following embryo numbers per genotype: E14.5 (*n*=4), E15.5 (*n*=6), E16.5 (*n*=4), E17.5 (*n*=4).

Next, we tracked growth and maturation of the hypertrophic zone between E14.5 and E17.5 in more detail by monitoring the length of the COL2 and COLX protein localization regions in control and *Prx1-cre;Foxc1*^Δ/Δ^*;Foxc2*^Δ/Δ^ embryos. No differences in the length of COL2-expressing regions were observed between the control and mutant limb sections at all time points examined (Fig. S3A-P). Moreover, the distance between the RZ and HCs was not affected in the *Prx1-cre;Foxc1*^Δ/Δ^*;Foxc2*^Δ/Δ^ embryos, indicating that the IHH-PTHLH signaling network was functioning. The size of the COLX-expressing domain was reduced at E14.5 and E15.5 in *Prx1-cre;Foxc1*^Δ/Δ^*;Foxc2*^Δ/Δ^ tibias (Fig. 6A-D,I). At later ages expanded proximal and distal COLX-expressing HC regions were seen at E16.5 and E17.5 compared with the control limbs (Fig. 6E-I). In addition, the POC, located between the two extended HC domains, was much smaller in *Prx1-cre;Foxc1*^Δ/Δ^*;Foxc2*^Δ/Δ^ limbs compared with the widely formed POC in control limbs at E16.5 and E17.5 (Fig. 6E-H,J). Although the length of the POC at E17.5 in *Prx1-cre;Foxc1*^Δ/Δ^*;Foxc2*^Δ/Δ^ embryos was comparable with the E16.5 POC in control embryos, indicating that formation of this region was slowed. These data suggest that in *Prx1-cre;Foxc1*^Δ/Δ^*;Foxc2*^Δ/Δ^ embryos, progression through chondrocyte differentiation is slowed, resulting in a reduction in the number of cells becoming hypertrophic at E14.5 followed by slowing through hypertrophy, leading to the formation of a smaller POC.

To test whether the expansion of the HC zone at E16.5 in *Prx1-cre;Foxc1*^Δ/Δ^*;Foxc2*^Δ/Δ^ mice was due to a reduction in cell death, we performed terminal deoxynucleotidyl transferase dUTP nick end labeling (TUNEL) *in situ* hybridization (ISH) at E15.5 and E16.5. At E15.5, TUNEL signal was detected in a population of cells adjacent to the perichondrium bordering the HCs in both control and mutant limbs (Fig. S4A,B, arrow). Cell death was not detected within the growth plate chondrocytes (Fig. S4A,B, yellow asterisk). At later stages, TUNEL-positive cells were found among the perichondrium and primary POC (Fig. S4C, green asterisk). However, in *Prx1-cre;Foxc1*^Δ/Δ^*;Foxc2*^Δ/Δ^ mutant limbs, cell death was primarily limited to the perichondrium and detected in only a small region of the nascent POC (Fig. S4D, green asterisk). As very few TUNEL-positive cells were detected in the HC in both the control and mutant tibia we cannot conclude that the changes in the length of the HC zone we observed in the *Prx1-cre;Foxc1*^Δ/Δ^*;Foxc2*^Δ/Δ^ embryos was a consequence of altered cell death in this region.

**Fig. 7.**
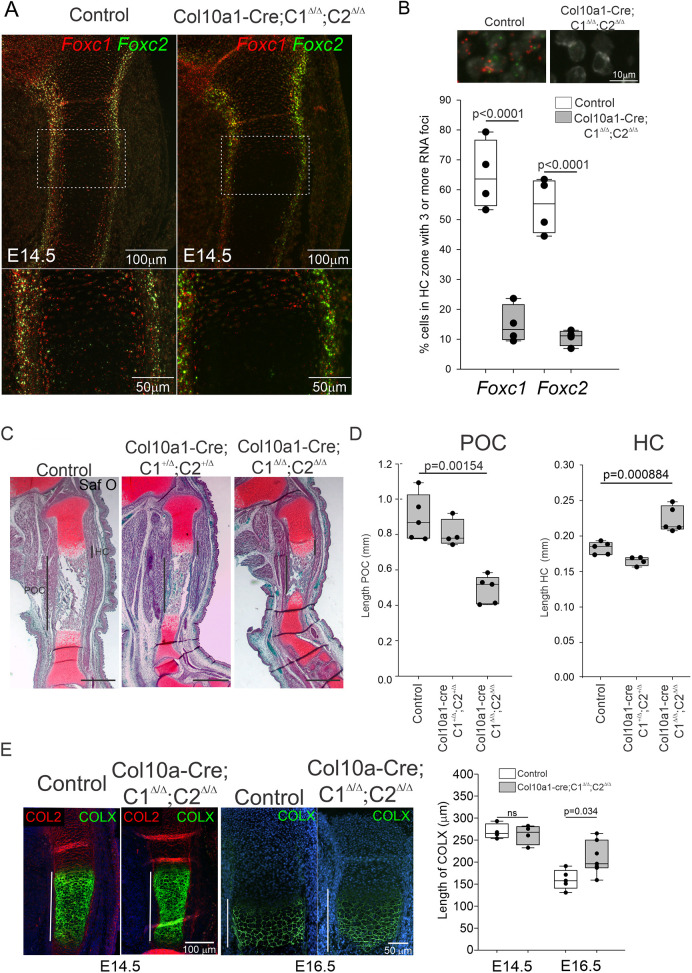
**FOXC1 and FOXC2 function in hypertrophic chondrocytes to regulate length of the primary ossification center.** (A) Expression of *Foxc1* (red) and *Foxc2* (green) mRNA in the growth plate was assessed at E14.5 in control and *Col10a1;Foxc1*^Δ/Δ^*;Foxc2*^Δ/Δ^ embryos. (B) The percentage of cells containing three or more foci for *Foxc1* and *Foxc2* expression was determined in control and *Col10a1;Foxc1*^Δ/Δ^*;Foxc2*^Δ/Δ^ embryos (*n*=4 embryos/genotype). Statistical analysis was performed with one-way ANOVA. (C) Safranin O histology of E16.5 tibia sections from control, *Col10a1;Foxc1*^Δ/Δ^*;Foxc2*^Δ/Δ^ and *Col10a1-cre;Foxc1^+^*^/Δ^*;Foxc2^+^*^/Δ^ embryos. Scale bars: 500 μm. (D) Length of the primary ossification center (POC) and hypertrophic chondrocyte (HC) zone from the proximal tibia was measured from control (*n*=5), *Col10a1-cre;Foxc1*^Δ/Δ^*;Foxc2*^Δ/Δ^ (*n*=5) and compound heterozygote *Col10a1;Foxc1^+^*^/Δ^*;Foxc2^+^*^/Δ^ (*n*=4) embryos at E16.5. Statistical analysis was performed using one-way ANOVA. (E) COLX immunofluorescence localization at E14.5 and E16.5 in the tibia of control or *Col10a1;Foxc1*^Δ/Δ^*;Foxc2*^Δ/Δ^ embryos. Statistical analysis was performed using one-way ANOVA. Box plots show the median value and the 25th and 75th percentile. Whiskers indicate the 10th and 90th percentile. Dots represent individual data points.

The expanded HC zone observed in the *Prx1-cre;Foxc1*^Δ/Δ^*;Foxc2*^Δ/Δ^ embryos at E16.5 and E17.5 may result from delays in progression through chondrocyte differentiation as a result of deleting *Foxc1* and *Foxc2* in either the early *Prx1*-expressing progenitor cells (thus slowing chondrogenesis), in the perichondrium that flanks the HC and POC, and/or the HCs themselves. To test this latter idea, we deleted *Foxc1* and *Foxc2* in HCs using the Bac-*Col10a1-cre* mouse (Gebhard et al., 2008; Park et al., 2015; referred to as *Col10a1-cre* hereafter). At E14.5, although very few Foxc1- or Foxc2-expressing cells could be detected in the hypertrophic zone of *Col10a1-cre;Foxc1*^Δ/Δ^*;Foxc2*^Δ/Δ^ embryos, expression of Foxc1 and Foxc2 was not affected in the growth plate or in the perichondrium of these embryos (Fig. 7A,B). At E16.5 the overall size of the tibia was shorter than the control embryos, with a reduced length of the POC and expansion in the HC zone (Fig. 7C,D). The lengths of the POC and HC zone in *Col10a1-cre;Foxc1^+^*^/Δ^*;Foxc2^+^*^/Δ^ embryos were similar to those of control animals. COLX IF analysis also confirmed an expanded HC zone in the *Col10a1-cre;Foxc1*^Δ/Δ^*;Foxc2*^Δ/Δ^ embryos at E16.5, but no changes in COLX expression were detected at E14.5 (Fig. 7E). Together these findings indicate that Foxc1 and Foxc2 function in HCs in a cell-autonomous manner to regulate the transition through chondrocyte hypertrophy.

### Impaired mineralization and bone formation in the POC of *Prx1-cre;Foxc1*^Δ/Δ^*;Foxc2*^Δ/Δ^ embryos

**Fig. 8.**
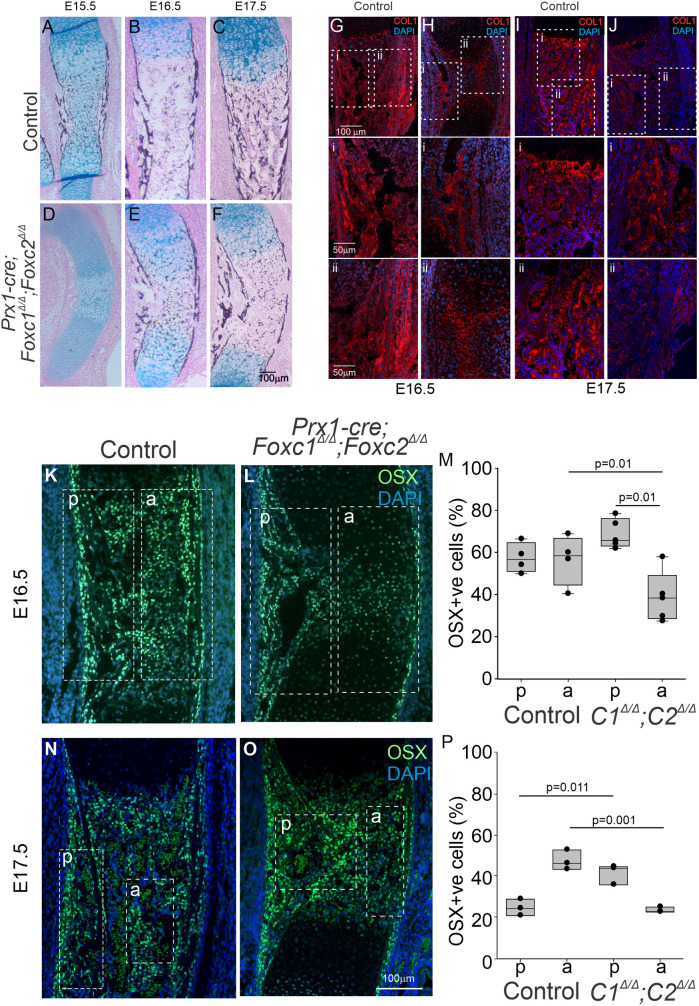
**Impaired mineralization and osteoblast localization in the POC of *Prx1-cre;Foxc1*^Δ/Δ^*;Foxc2*^Δ/Δ^ embryos.** (A-F) Mineralization was assessed in tibia sections by Von Kossa staining at E15.5 (A,D), E16.5 (B,E) and E17.5 (C,F) in the bone collar and the POC of control and *Prx1-cre;Foxc1*^Δ/Δ^*;Foxc2*^Δ/Δ^ embryos (*N*=3). (G-Jii) Collagen 1 (red) localization in the tibia POC was assessed in control (G,I) and *Prx1-cre;Foxc1*^Δ/Δ^*;Foxc2*^Δ/Δ^ (H,J) embryos at E16.5 and E17.5. (K-P) Osteoblast localization was determined through Osterix immunofluorescence signal in tibia sections at E16.5 and E17.5 in control and *Prx1-cre;Foxc1*^Δ/Δ^*;Foxc2*^Δ/Δ^ embryos. The entire POC was divided into posterior (p) and anterior (a) regions as indicated by white dashed boxes. The percentage of OSX-positive cells versus DAPI-positive cells was determined. Statistical analysis was determined by one-way ANOVA. Box plots show the median value and the 25th and 75th percentile. Whiskers indicate the 10th and 90th percentile. Dots represent individual data points.

We next examined osteoblast formation and bone mineralization in *Prx1-cre;Foxc1*^Δ/Δ^*;Foxc2*^Δ/Δ^ embryos. First, we monitored mineralization of the tibia using Von Kossa staining. In the control embryos we detected a strong signal in the tibia bone collar starting at E15.5 (Fig. 8A). In the presumptive POC, mineralized chondrocytes remained present at E15.5, began to be replaced at E16.5 and ultimately formed the POC at E17.5 (Fig. 8A-C). In the *Prx1-cre;Foxc1*^Δ/Δ^*;Foxc2*^Δ/Δ^ embryos, no mineralization was detected in the tibia bone collar or the HCs at E15.5 (Fig. 8D). At E16.5, bone collar mineralization was detected and mineralized chondrocytes were persistent in the hypertrophic zone of *Prx1-cre;Foxc1*^Δ/Δ^*;Foxc2*^Δ/Δ^ embryos (Fig. 8E). By E17.5, *Prx1-cre;Foxc1*^Δ/Δ^*;Foxc2*^Δ/Δ^ embryos displayed a strong Von Kossa signal in the bone collar with little mineralization detected in the POC (Fig. 8F). Prominent COL1 signal was localized throughout the POC of control embryos in a pattern expected for the osteoid at E16.5 and E17.5 (Fig. 8G,I), whereas in the *Prx1-cre;Foxc1*^Δ/Δ^*;Foxc2*^Δ/Δ^ embryos COL1 localization was detected in osteoid-like structures in posterior bone collar. In the center/anterior of the tibia, COL1 was localized in a meshwork pattern reminiscent of the ECM of HCs in *Prx1-cre;Foxc1*^Δ/Δ^*;Foxc2*^Δ/Δ^ embryos (compare Fig. 8H,J, panel i versus ii). OSX-positive cells also displayed an asymmetric localization in the developing tibia POC of *Prx1-cre;Foxc1*^Δ/Δ^*;Foxc2*^Δ/Δ^ embryos (Fig. 8K-P). Abundant OSX-positive cells were detected in the posterior bone collar of both control and mutant embryos at E16.5 (Fig. 8K-M) and E17.5 (Fig. 8N-P), whereas a lower density of OSX-positive cells was detected in the newly forming POC at E16.5 and E17.5 in the *Prx1-cre;Foxc1*^Δ/Δ^*;Foxc2*^Δ/Δ^ embryos. Collectively these data reveal that osteoblast formation and mineralization does occur in the bone collar of *Prx1-cre;Foxc1*^Δ/Δ^*;Foxc2*^Δ/Δ^ embryos. However, these osteoblast progenitor cells may not be able to populate the center of the newly forming POC as efficiently as in controls, resulting in reduced numbers of OSX-positive osteoblasts and impaired mineralization of the POC.

### Bone remodeling was compromised by the absence of *Foxc1* and *Foxc2* in long bones

**Fig. 9.**
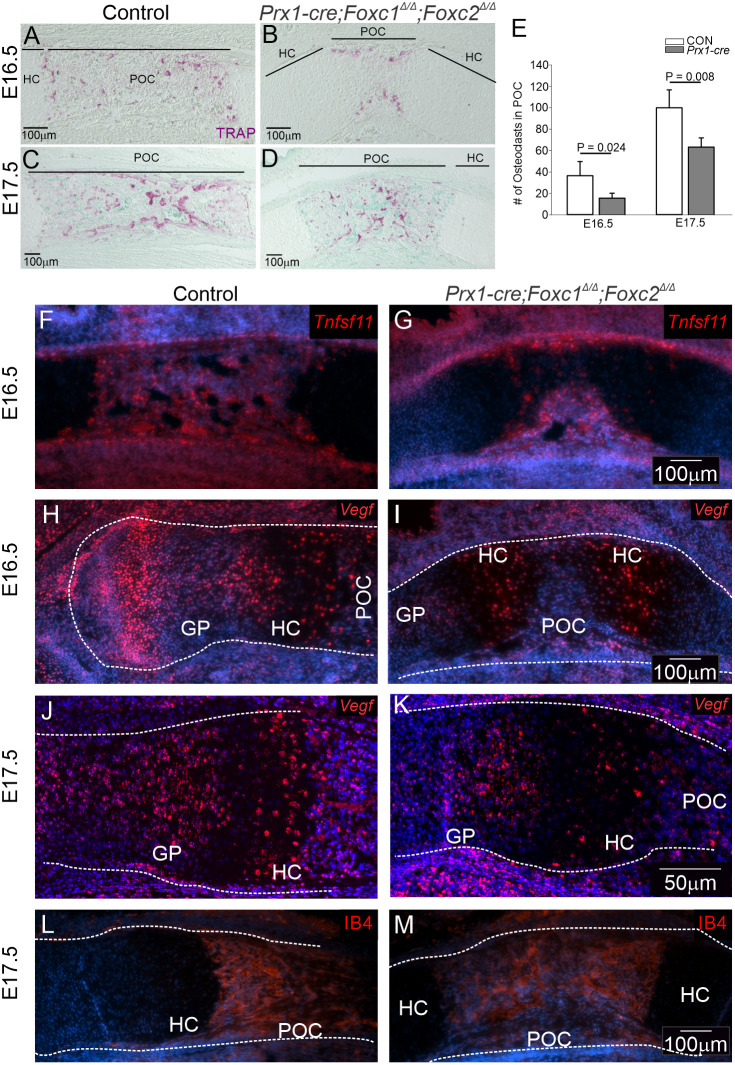
**Reduced osteoclast activation in the POC of *Prx1-cre;Foxc1*^Δ/Δ^*;Foxc2*^Δ/Δ^ embryos.** (A-D) TRAP staining was performed on E16.5 (A,B) and E17.5 (C,D) control and *Prx1-cre;Foxc1*^Δ/Δ^*;Foxc2*^Δ/Δ^ tibia to assess osteoclast localization in the POC. Widespread TRAP signal was distributed throughout the POC of control embryos (A). In contrast, TRAP signal was localized at the bone collar in mutants (B). At E17.5, osteoclasts had a comparable distribution pattern throughout the POC in both control and mutant limbs (C,D). (E) Fewer TRAP-positive cells were detected in the *Prx1-cre;Foxc1*^Δ/Δ^*;Foxc2*^Δ/Δ^ limb at E16.5 and E17.5 in comparison with their controls. (F-M) Expression of *Tnfsf11* (RANKL) mRNA was detected in the control and *Prx1-cre;Foxc1*^Δ/Δ^*;Foxc2*^Δ/Δ^ POC (F,G). *Vegfa* (*Vegf*) mRNA was localized in the HC and POC in control and *Prx1-cre;Foxc1*^Δ/Δ^*;Foxc2*^Δ/Δ^ limbs at E16.5 (H,I) and E17.5 (J,K). IB4 was detected in both E17.5 control and *Prx1-cre;Foxc1*^Δ/Δ^*;Foxc2*^Δ/Δ^ POC (L,M). The bone anlage is outlined with dashed lines. GP, growth plate; HC, hypertrophic chondrocytes; POC, primary ossification center. Statistical analysis was performed by the Student's *t*-test. Error bars indicate standard deviation. Data presented are representative of three littermate pairs.

We next examined whether impaired bone remodeling and/or vascularization prevented invasion of osteoblasts from the bone collar into the interior of the *Prx1-cre;Foxc1*^Δ/Δ^*;Foxc2*^Δ/Δ^ limbs. We visualized osteoclasts using Tartrate-Resistant Acid Phosphatase (TRAP) staining at E16.5 and E17.5 in the control and *Prx1-cre;Foxc1*^Δ/Δ^*;Foxc2*^Δ/Δ^ limbs. TRAP-positive cells were localized in the perichondrium, periosteum and the osteochondral junction in the control tibia POC. However, there were fewer osteoclasts in the *Prx1-cre* mutants at both time points (Fig. 9A-E). The low number of osteoclasts in the mutants may cause slower bone resorption activity leading to reduced bone remodeling and formation of shorter limbs (Lademann et al., 2020)*.* We then monitored expression of *Tnfsf11* (RANKL) which stimulates osteoclast recruitment. Expression of *Tnfsf11* was detected in control and *Prx1-cre;Foxc1*^Δ/Δ^*;Foxc2*^Δ/Δ^ mutant limbs, although the expression region was much smaller in the mutant limbs, likely owing to the reduction of osteoblast formation (Fig. 9F,G). Angiogenesis and blood vessel invasion into the POC are necessary for bone formation (Sivaraj and Adams, 2016). Thus, we assessed whether vascularization was affected in the limbs of *Prx1-cre;Foxc1*^Δ/Δ^*;Foxc2*^Δ/Δ^ embryos. *Vegfa* mRNA was localized in the HCs in both control and the *Prx1-cre* mutant limbs, with more *Vegfa* signal marking the extended HC zone in the *Prx1-cre;Foxc1*^Δ/Δ^*;Foxc2*^Δ/Δ^ E16.5 tibia (Fig. 9H,I). Moreover, vascular endothelial marker isolectin-B4 (IB4) localization confirmed the presence of blood vessels in both control and mutant POCs (Fig. 9L,M) with *Vegfa* mRNA expression detected in the GP and HC at E17.5 (Fig. 9J,K). Collectively, these results suggest that expression of *Foxc1/2* transcription factors in limb bud mesenchyme plays a role in facilitating osteoclast recruitment and activation, which is needed for osteoblast function and mineralization of the POC. In contrast, deletion of *Foxc1* and *Foxc2* in limb bud mesenchymal cells did not block blood vessel invasion into the bone cavity.

### *Foxc1* is required for *Phex* expression to maintain bone mineralization

**Fig. 10.**
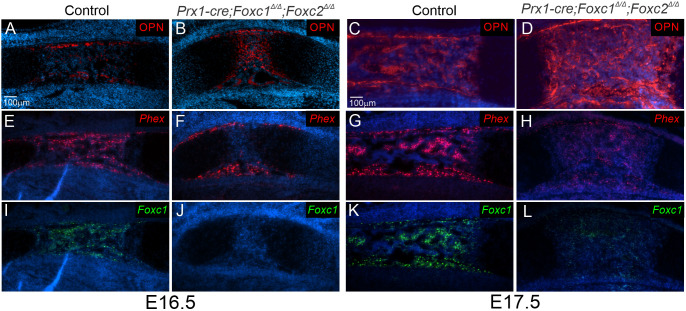
**FOXC1 and FOXC2 are required for *Phex* expression in the POC.** (A-D) OPN levels were determined in the POC of the control and *Prx1-cre;Foxc1*^Δ/Δ^*;Foxc2*^Δ/Δ^ tibia at E16.5 (A,B) and E17.5 (C,D). (E-H) *Phex* mRNA expression was determined by *in situ* hybridization at E16.5 (E,F) and E17.5 (G,H) in control and *Prx1-cre;Foxc1*^Δ/Δ^*;Foxc2*^Δ/Δ^ tibia. (I-L) *Foxc1* mRNA expression was determined by *in situ* hybridization at E16.5 (I,J) and E17.5 (K,L) in the control and mutant tibia. Data were obtained from analysis of three control and mutant littermate pairs.

OPN (SPP1) protein is detected in the bone collar and the POC in both control and *Prx1-cre;Foxc1*^Δ/Δ^*;Foxc2*^Δ/Δ^ limbs (Fig. 10A-D). However, there was a more intense OPN signal located within the smaller POC of mutant limbs at E16.5 and E17.5 (Fig. 10B,D). In order for mineralization to proceed, OPN is proteolytically processed and degraded by enzymes such as phosphate regulating endopeptidase homolog X-linked (PHEX; Addison et al., 2010; Zurick et al., 2013). We previously found that *Phex* mRNA levels were reduced in *Col2-cre;Foxc1*^Δ/Δ^*;Foxc2*^Δ/Δ^ mice (Almubarak et al., 2021). Consistent with this, we found a dramatic reduction in *Phex* expression in the POC of *Prx1-cre;Foxc1*^Δ/Δ^*;Foxc2*^Δ/Δ^ tibias at E16.5 and E17.5 compared with controls (Fig. 10E-L). In addition, areas where *Phex* expression was reduced in *Prx1-cre;Foxc1*^Δ/Δ^*;Foxc2*^Δ/Δ^ limbs overlapped with areas where OPN levels were elevated. These results indicated that *Foxc1* and *Foxc2* are required for the expression of *Phex* in the POC. The consequent absence of *Phex* expression may lead to improper processing of OPN and thus block the proper mineralization in the POC of *Prx1-cre;Foxc1*^Δ/Δ^*;Foxc2*^Δ/Δ^ mutants.

## DISCUSSION

To understand how FOXC1 and FOXC2 function in early steps of endochondral ossification, we generated two conditional compound knockouts that target skeletogenic progenitors in the entire skeleton and limb bud (*Sox9-cre* or *Prx1-cre*, respectively). Loss of FOXC1 and FOXC2 function in the *Sox9* lineage resulted in the near absence of chondrogenesis in the vertebral column and a reduction in the size of the cartilaginous elements in the limb buds at E12.5. This difference in the effect of FOXC1/FOXC2 loss in *Sox9*-expressing cells in the axial versus the appendicular skeleton may reflect either distinct roles for FOXC1 and FOXC2 in these two regions of the embryo; or a compensation by other transcription factors that are restricted to the appendicular skeleton. Nevertheless, Foxc proteins are important regulators of limb skeletogenesis, as *Prx1-cre;Foxc1*^Δ/Δ^*;Foxc2*^Δ/Δ^ embryos displayed smaller malformed fore- and hindlimbs. Limb defects were more evident in distal bone elements compared with proximal ones. For example, in the hindlimb, cartilage formation and bone mineralization were markedly reduced in the tarsals, metatarsals and phalanges of *Prx1-cre;Foxc1*^Δ/Δ^*;Foxc2*^Δ/Δ^ embryos, and the tibia did form but was shorter and curved and the fibula was grossly truncated. In contrast, the femur was shorter, but did not appear to be noticeably different from control limbs. We propose that the proximal-distal patterning effects we observe are a result of a disruption of the temporal sequence of limb development rather than a spatial patterning effect. Limb structures develop in a proximal-to-distal manner such that bones in the stylopod are specified and form before the zeugopod and autopod elements (Tickle and Towers, 2020). One explanation is that fewer skeletogenic cells are available in *Prx1-cre;Foxc1*^Δ/Δ^*;Foxc2*^Δ/Δ^ embryos, either through impaired differentiation of precursor cells into chondrocytes or reduced proliferation of the progenitor population, and thus this pool of cells is exhausted or diminished as distal structures form. Indeed, we demonstrated elevated expression of *Foxc1* and *Foxc2* mRNAs at the onset of condensation in the limb bud mesenchyme and also in immature chondrocytes. As limb development proceeds, expression of *Foxc1* and *Foxc2* mRNAs is decreased in the growth plate and confined to the surrounding perichondrium (Fig. 2; Almubarak et al., 2021). Furthermore, the grossly impaired chondrocyte formation observed in *Sox9^ires-Cre/+^;Foxc1*^Δ/Δ^*;Foxc2*^Δ/Δ^ embryos (Fig. S1) supports a prominent role for Foxc factors in the early stages of chondrogenesis. We also noted that *Prx1-cre;Foxc1*^Δ/Δ^*;Foxc2*^Δ/Δ^ limbs exhibited reduced bone formation in the posterior zeugopod cartilage elements compared with the anterior elements. In the forelimbs, the ulna was underdeveloped compared with the radius, and in the hindlimbs, the fibula was smaller than the tibia. No differences in *Foxc1* or *Foxc2* expression have been observed between anterior and posterior zeugopod elements (Almubarak et al., 2021). Fate-mapping of Msx1-expressing forelimb bud precursor cells has indicated that an influx of Msx1-expressing limb bud mesenchymal precursor cells into the posterior zeugopod cartilage element (i.e. the ulna) occurs before that of the anterior zeugopod cartilage element (i.e. the radius; Markman et al., 2023). Perhaps this difference in the timing of the formation and differentiation of the anterior versus the posterior zeugopod cartilage elements renders the posterior zeugopod elements, with a more limited temporal window to support their formation, more sensitive to *Foxc1*/*2* loss. We also observed that the growth of the autopod cartilage elements was severely decreased in *Prx1-cre;Foxc1*^Δ/Δ^*;Foxc2*^Δ/Δ^ embryos, but that patterning of these elements was by and large normal. Differentiation of skeletal elements in the limb occurs through a complex three-dimensional process, during which different pools of progenitor cells give rise to specific cartilage elements (e.g. radius versus ulna; Markman et al., 2023). Thus, the varying kinetics of precursor cell influx and cartilage differentiation of these differing cartilage elements may render these elements differentially affected by loss of *Foxc1* and *Foxc2*.

The progression through chondrocyte differentiation was slowed in *Prx1-cre;Foxc1*^Δ/Δ^*;Foxc2*^Δ/Δ^ embryos and was manifested as fewer cells expressing *Ihh* mRNA and COLX protein at E13.5-E14.5 when formation of the PHC and HCs normally occur. We observed an initial smaller hypertrophic zone with lower levels of COLX production and reduced expression of *Ihh* in PHCs (Fig. 4 and Fig. S2). Although fewer IHH-expressing cells were produced at E14.5 in *Prx1-cre;Foxc1*^Δ/Δ^*;Foxc2*^Δ/Δ^ embryos, the ability of IHH to signal was not affected (as evidenced by the activation of *Ptch1*/*2* expression). The smaller region of IHH-producing cells in *Prx1-cre;Foxc1*^Δ/Δ^*;Foxc2*^Δ/Δ^ embryos at E14.5 alters the spatial organization and partitioning of the IHH-PTHLH signal. In *Prx1-cre;Foxc1*^Δ/Δ^*;Foxc2*^Δ/Δ^ embryos, expression of *Pthlh* and *Pth1r* at E14.5 is displaced towards the HC and newly forming POC, whereas these genes are localized more distally in control embryos. As PTHLH functions to inhibit chondrocyte hypertrophy, this centralized concentration of PTHLH signaling in HC of *Prx1-cre;Foxc1*^Δ/Δ^*;Foxc2*^Δ/Δ^ embryos may contribute to the reduced *Ihh* expression and slow further HC formation. As the cartilage element grows, separation of IHH-PTHLH signaling occurs and *Ihh* expression levels are restored in E16.5 *Prx1-cre;Foxc1*^Δ/Δ^*;Foxc2*^Δ/Δ^ embryos.

**Fig. 11.**
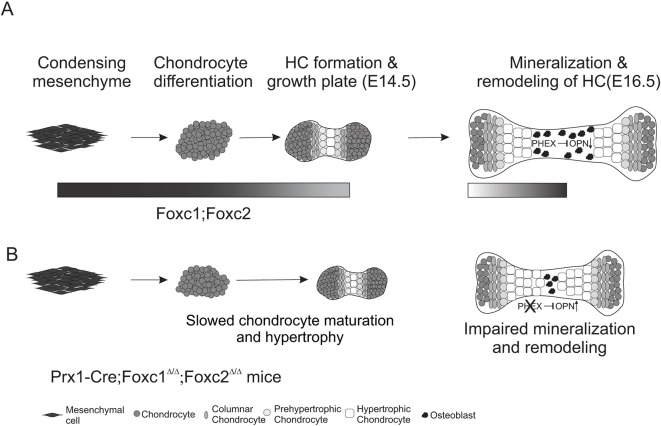
**FOXC1 and FOXC2 function at two separate phases in endochondral ossification of the limb.** We propose that FOXC1 and FOXC2 act at different stages of endochondral ossification of the limb skeleton (depicted by shaded bar). First, these factors function in the differentiation of limb bud mesenchyme towards the chondrocyte lineage. Here, FOXC1 and FOXC2 regulate chondrocyte proliferation and maturation towards hypertrophic chondrocytes. In *Prx1-cre;Foxc1*^Δ/Δ^*;Foxc2*^Δ/Δ^ mutants we observed fewer *Ihh*-expressing prehypertrophic chondrocytes and hypertrophic chondrocytes. Second, once IHH expression is established, the growth plate is organized and hypertrophic chondrocytes have formed (after E14.5), FOXC1 and FOXC2 may not be required in differentiating chondrocytes at this stage. Instead, after E14.5 *Foxc1* and *Foxc2* may function to regulate remodeling and removal of the hypertrophic chondrocytes to create the POC. At this stage FOXC1 and FOXC2 are required for *Phex* expression, which functions with OPN to allow osteoblast mineralization to occur.

We propose that *Foxc1* and *Foxc2* function in the progression of the early chondrocytes formed at mesenchymal condensations towards chondrocyte hypertrophy. It is important to consider that the chondrocytes that form shortly after mesenchyme condensation will differentiate towards chondrocyte hypertrophy without the influence of IHH produced in PHC and HCs. FOXC1 and FOXC2 may function in this initial progression of chondrocyte differentiation and have less of an impact on chondrocyte maturation once HCs are formed (Fig. 11). In addition to slowing the progression of chondrocyte differentiation towards hypertrophy, the loss of *Foxc1*/*2* also delays the endochondral ossification of HCs. As FOXC1 can directly regulate COLX expression in chondrocytes *in vitro* (Yoshida et al., 2015), it is possible that FOXC1 (and presumably FOXC2) may regulate the initial formation of the HC zone. Consistent with this idea is the dramatically reduced expression of *Col10a1* mRNA and protein we observe at E13.5 and E14.5 in the tibia (Fig. 6 and Fig. S2). Once HCs are formed, additional transcription factors such as Foxa family members may function in place of FOXC1 and FOXC2 to regulate chondrogenesis once the growth plate forms and the HC zone is established (Ionescu et al., 2012).

Loss of FOXC1 and FOXC2 function delayed the terminal stages of chondrocyte hypertrophy and dysregulated HC remodeling. We observed expanded HC zones and smaller POCs from E16.5 onward in *Prx1-cre;Foxc1*^Δ/Δ^*;Foxc2*^Δ/Δ^ embryos. TRAP staining was reduced in the POC of *Prx1-cre;Foxc1*^Δ/Δ^*;Foxc2*^Δ/Δ^ embryos, suggesting that the expansion of the HC zone may result from impaired remodeling of the HCs. We demonstrate that FOXC1 and FOXC2 functions in HC and the elongated hypertrophic zone observed was not solely a result of the slowed progression of chondrocyte differentiation. Although *Col10a1-cre* conditional mutants resulted in an expanded HC zone and smaller POC, we did not observe any bowing of bones or other malformations observed in the *Prx1-cre;Foxc1*^Δ/Δ^*;Foxc2*^Δ/Δ^ embryos. These differences may result from FOXC1 and FOXC2 functioning in cell lineages targeted by *Prx1-cre*, such as the osteoblast progenitors in the perichondrium/periosteum that are not in *Col10a1-cre* conditional mutants. What functions FOXC1 and FOXC2 play in HCs is undetermined. These factors may regulate recruitment chondroclasts needed to remodel HC and form the POC, or may participate in HC-to-osteoblast differentiation events to populate the POC.

Mineralization of the POC was affected in *Prx1-cre;Foxc1*^Δ/Δ^*;Foxc2*^Δ/Δ^ embryos. FOXC1 and FOXC2 are required for osteoblast differentiation *in vitro* (Hopkins et al., 2016; Mirzayans et al., 2012; Park et al., 2011; Rice et al., 2003); however, impaired osteoblast differentiation alone likely does not account for the phenotypes we observe. In the perichondrium/periosteum surrounding the tibia in *Prx1-cre;Foxc1*^Δ/Δ^*;Foxc2*^Δ/Δ^ embryos, OSX-positive osteoblasts were present from E15.5 onward, although mineralization was delayed. In the presumptive POC of *Prx1-cre;Foxc1*^Δ/Δ^*;Foxc2*^Δ/Δ^ embryos, OSX-expressing osteoblasts formed but fewer were detected. Osteoblast progenitors from the bone collar invade into the POC along with blood vessels. Vascularization of the POC did not appear to be affected as HCs expressed *Vegfa* and blood vessels were present in the *Prx1-cre;Foxc1*^Δ/Δ^*;Foxc2*^Δ/Δ^ POC (Gerber et al., 1999; Zelzer et al., 2004). However, as reduced OSX-labeled cells were detected in the POC of *Prx1-cre;Foxc1*^Δ/Δ^*;Foxc2*^Δ/Δ^ embryos, it is possible that *Foxc1/2-*deficient osteoblasts are unable to associate with vascular endothelial cells when blood vessels populate the POC. We did observe persistent OPN localization and dramatically reduced expression of *Phex* in the POC of *Prx1-cre;Foxc1*^Δ/Δ^*;Foxc2*^Δ/Δ^ embryos. As PHEX acts to proteolytically process OPN for mineralization to proceed (Addison et al., 2010; Barros et al., 2013), loss of *Phex* expression in *Prx1-cre;Foxc1*^Δ/Δ^*;Foxc2*^Δ/Δ^ leads to an accumulation of OPN that disrupts mineralization in the POC. Whether FOXC1 and FOXC2 directly bind to and regulate *Phex* expression is a current research question that we are exploring.

### Limitations

We focused our analysis on double homozygous mutant embryos and thus we were not able to reach conclusions on any specific roles for either FOXC1 or FOXC2 in regulating endochondral ossification. In addition, we directed our analysis to embryonic development as *Prx1-cre;Foxc1*^Δ/Δ^*;Foxc2*^Δ/Δ^ mice die shortly after birth and thus the postnatal functions for FOXC1 and FOXC2 are yet to be determined.

In summary we report the overlapping roles for FOXC1 and FOXC2 in limb bud mesenchymal progenitors to regulate endochondral ossification in the appendicular skeleton (Fig. 11). We propose that FOXC1 and FOXC2 function at two phases in endochondral ossification: during the maturation of chondrocyte progenitors towards the formation of the hypertrophic zone, and later in the remodeling of HCs to allow formation of the POC and marrow space.

## MATERIALS AND METHODS

### Mouse models

Experiments using mouse models were either approved by the University of Alberta Animal Care and Use Committee (AUP804) or approved by the Harvard Medical School Institutional Animal Care and Use Committee (IACUC). To explore whether FOXC1 and FOXC2 share overlapping roles in Sox9-expressing cells, *Foxc1^fl/fl^;Foxc2^fl/fl^* (Sasman et al., 2012) mice were mated with mice containing *Sox9^ires-Cre/+^* (Akiyama et al., 2005) to generate E12.5 embryos which either deleted neither, one or both of these Foxc family members.

*Prx1-cre;Foxc1*^Δ/Δ^*;Foxc2*^Δ/Δ^ mice were generated through crossing *Foxc1^fl/fl^;Foxc2^fl/fl^* (Sasman et al., 2012) with *Prx1-cre*^+/−^ mice (Logan et al., 2002). Timed pregnancies were performed by crossing male *Prx1-cre^+/−^;Foxc1^+/fl^;Foxc2^+/fl^* mice to female *Foxc1^fl/fl^;Foxc2^fl/fl^* mice. *Col10a1-cre;Foxc1*^Δ/Δ^*;Foxc2*^Δ/Δ^ mice (Gebhard et al., 2008; Park et al., 2015) were generated using a similar strategy. The day of the detection of a vaginal plug was denoted as E0.5. We genotyped weaned mice using ear notch biopsies and embryos using skin. The genotyping process was conducted using the KAPA mouse genotyping kit (Millipore Sigma) using the following primer pairs: *Foxc1* (forward 5′-ATTTTTTTTCCCCCTACAGCG-3′; reverse 5′-ATCTGTTAGTATCTCCGGGTA-3′), *Foxc2* (forward 5′-CTCCTTTGCGTTTCCAGTGA-3′; reverse 5′-ATTGGTCCTTCGTCTTCGCT-3′) and *Prx1-cre* (forward 5′-GCCTGCATTACCGGTCGATGCAACGA-3′; reverse 5′-GTGGCAGATGGCGCGGCAACACCATT-3′). All experimental comparisons were made between littermates, with a minimum of three litters investigated per experiment, unless otherwise noted.

### Whole skeleton staining

Embryos were collected at E12.5 and E18.5 and processed for whole skeleton Alcian Blue and Alizarin Red staining as described in Rigueur and Lyons (2014).

### Tissue preparation

Tissues were dissected at specific stages and fixed in 4% paraformaldehyde at 4°C overnight before embedding in paraffin. Sections were cut at 5 μm thickness and collected on Superfrost-plus slides (Thermo Fisher Scientific). E17.5 limbs were either embedded after fixation or decalcified with EDTA at 4°C overnight before paraffin embedding.

### Histology

All sections were first dewaxed with xylene and rehydrated with graded ethanol and water. For Safranin O staining, sections were stained with Hematoxylin for 8 min and rinsed with running tap water for 10 min. Next, sections were stained with 0.001% Fast Green for 5 min, followed with 1% acetic acid wash for 10-15 s to stabilize the staining. Slides were then stained with 0.1% Safranin O for 5 min, rehydrated with 100% ethanol and xylene, and mounted with coverslips. For Alcian Blue-Von Kossa staining, sections were incubated with 1% silver nitrate solution under ultraviolet (UV) light for 20 min. Slides were then rinsed with two water changes followed by 5 min incubation with 5% sodium thiosulfate to remove the unreacted silver. Sections were then stained with Alcian Blue for 30 min and counterstained with Nuclear Fast Red for 5 min. For TRAP staining, sections were then incubated in a pre-warmed TRAP Staining solution mix [50 mM sodium acetate, 22 mM L-(+) tartaric acid, 50 mM napthol AS-MX phosphate and 70 µM Fast Violet Red] at 37°C for 30 min. Next, slides were rinsed with water and counterstained with 0.02% Fast Green.

### *In situ* hybridization

Fluorescent ISH for multiplex was performed using RNA scope Multiplex Fluorescent kit following the manufacturer's protocol for paraffin embedded sections (Advanced Cell Diagnostics). Sections were boiled in antigen retrieval solutions for 15 min. The following probes were used: negative control (REF: 310043); *Foxc1* (REF: 412851); *Foxc2* (REF: 406011); Sox9 (REF: 401051-C3); *Fgfr3* (REF: 440771); *Fgfr1* (REF: 454941); *Ihh* (REF: 413091); *Gli1*(REF: 311001); *Gli2* (REF: 405771-C2); *Gli3* (REF: 445511); *Pthlh* (REF: 456521); *Pth1r* (REF: 426191); *Ptch1* (REF: 402811); *Ptch2* (REF: 435131); *Colx* (REF: 433491); *Tnfsf11* (REF: 410921); *Vegf* (REF: 436961); and *Phex* (REF: 426201).

### Immunofluorescence

Paraffin sections were collected and slides were prepared as described above. For SOX9, OSX, OPN and RUNX2 antibodies, antigen retrieval was performed through boiling the slides in citrate buffer (10 mM trisodium citrate, pH 6.0; 0.05% Tween 20) for 20 min. For COL1, COL2a, COLX, MMP13 and VEGFA antibodies, samples were incubated in hyaluronidase for 30 min at 37°C. Next, slides were blocked in 5% donkey serum in PBS with 0.05% Triton X-100 (PBSX) for 1 h. Slides were then incubated with the primary antibody overnight at 4°C. The following antibodies were used for immunofluorescence microscopy: COLIIa (Abcam, ab185430, 1:100); COLX (Abcam, ab58632, 1:50); COL I (Abcam, ab88147; GR3225500-1, 1:100); MMP13 (Abcam, ab39012, 1:100); RUNX2 (Abcam, ab76956, 1:200); IB4 (Thermo Fisher Scientific, VECTB1205, 1:500); SOX9 (Millipore Sigma, AB55535, 1:200); OPN (SCBT, sc22536-R, 1:100); OSX (SCBT, sc21742, 1:100).

### KI67 cell proliferation assay

Cell proliferation in the developing hindlimb was assessed using KI67 IF as described previously (Almubarak and Berry, 2022). The KI67 antibody (Bethyl Laboratories, IHC00075) was used at a concentration of 1:100. The region of interest included the growth plate bounded distally by the low cell density characteristic of the HCs as well as cells interior of the perichondrium. The perichondrial cells were identified as those cells oriented perpendicular to the growth plate cells.

### Quantitative reverse transcription PCR

RNA was collected from the hindlimbs of E13.5 embryos (*n*=4 per genotype) using RNeasy extraction kits (Qiagen). The stylopod/zeugopod regions were dissected away from the distinctive autopod paddle. Reverse transcription and qPCR reactions were performed as described previously (Almubarak et al., 2021). Expression was normalized to *Hprt* and *Rn18s* levels and analyzed with Maestro (Bio-Rad). Primers were purchased from Integrated DNA Technologies as predesigned primer pairs or were manually designed. Primer sequences are as follows: *Foxc1* (Mm.PT.56a.33593611.g); *Foxc2* (Mm.PT.58.33608703.g); *Col2a* (F 5′-CCGTCATCGAGTACCGATCA-3′; R 5′-CAGGTCAGGTCAGCCATTCA-3′); *Pthlh* (F 5′-CATCAGCTACTGCATGACAAGG-3′; R 5′-GGTGGTTTTTGGTGTTGGGAG-3′); *Fgfr3* (F 5′-GACACCAAAAGACCAAACATCA-3′; R 5′-GCACAACCTGGACTACTACAAG-3′); *Ihh* (Mm.PT.58.30489545); *Runx2* (F 5′-ACCATGGTGGAGATCATCG-3′; R 5′-TAACAGCGCAGGCATTTCG-3′); *Col10a1* (Mm.PT.58.28877219); *Hprt* (Mm.PT.39a.22214828); *Rn18s* (F 5′-AACGAGACTCTGGCATGCTAACT-3′; R 5′-CGCCACTTGTCCCTCTAAGAA-3′).

### TUNEL assay

Cell death was detected using the *In situ* Cell Death Detection Kit, TMR red (Roche). Tibia sections were obtained and processed as described above. Slides were permeabilized with proteinase K working solution (10 μg/ml in 10 mM Tris/HCL, pH 7.4-8) for 30 min at 37°C and washed twice with 1× PBS. Then, sections were treated with the TUNEL reaction mixture for 60 min in a humidified atmosphere at 37°C. Slides were then washed three times with 1× PBS and stained with DAPI for 5 min and mounted with Prolong Gold anti-fade reagent (Invitrogen).

### Statistical analysis and image quantification

Statistical analyses were conducted using SigmaPlot 13 using a minimum of four littermate pairs unless otherwise stated. One-way ANOVA and unpaired two-tailed Student's *t*-test were performed as indicated in the figure legends, with *P*<0.05 as a cut-off for statistical significance. Cell number quantification was obtained using ImageJ, and size of gene expression and protein localization domains were measured using ImageJ and CorelDRAW 2020. Experimenters were initially unaware to the genotype, however obvious phenotypic abnormalities in the *Prx1-cre;Foxc1*^Δ/Δ^*;Foxc2*^Δ/Δ^ embryos made it difficult to maintain this state. We assayed multiple sections per embryo to ensure we were comparing similar depths of field in control versus mutant embryos.

## Supplementary Material



10.1242/develop.202798_sup1 Supplementary information
