# Supplementary material for: FOXC1 and FOXC2 regulate growth plate chondrocyte maturation towards hypertrophy in the embryonic mouse limb skeleton
Source: Development. 2024 Aug 22;151(16):dev202798. doi: 10.1242/dev.202798 (PMC11361642; doi:10.1242/dev.202798)
Supplement: Supplementary information [file develop-151-202798-s1.pdf]

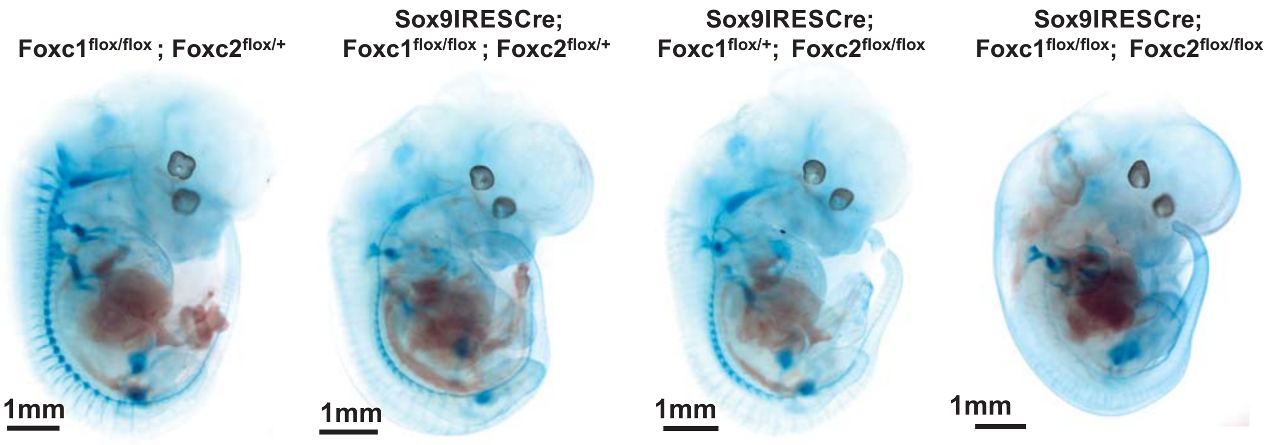

Fig. S1. ***Foxc1* and *Foxc2* share roles to promote axial chondrogenesis**.
Whole embryos littermates embryos at E12.5 were stained with Alcian blue to detect chondrogenic differentiation. Similar results have been obtained with embryos from 2 litters, containing 3 embryos of the least frequently occurring genotype (i.e., *Sox9*$^{ires\text{-}Cre/+}$*; Foxc1*$^{flox/flox}$*;Foxc2*$^{flox/flox}$*)*.

Development • Supplementary information

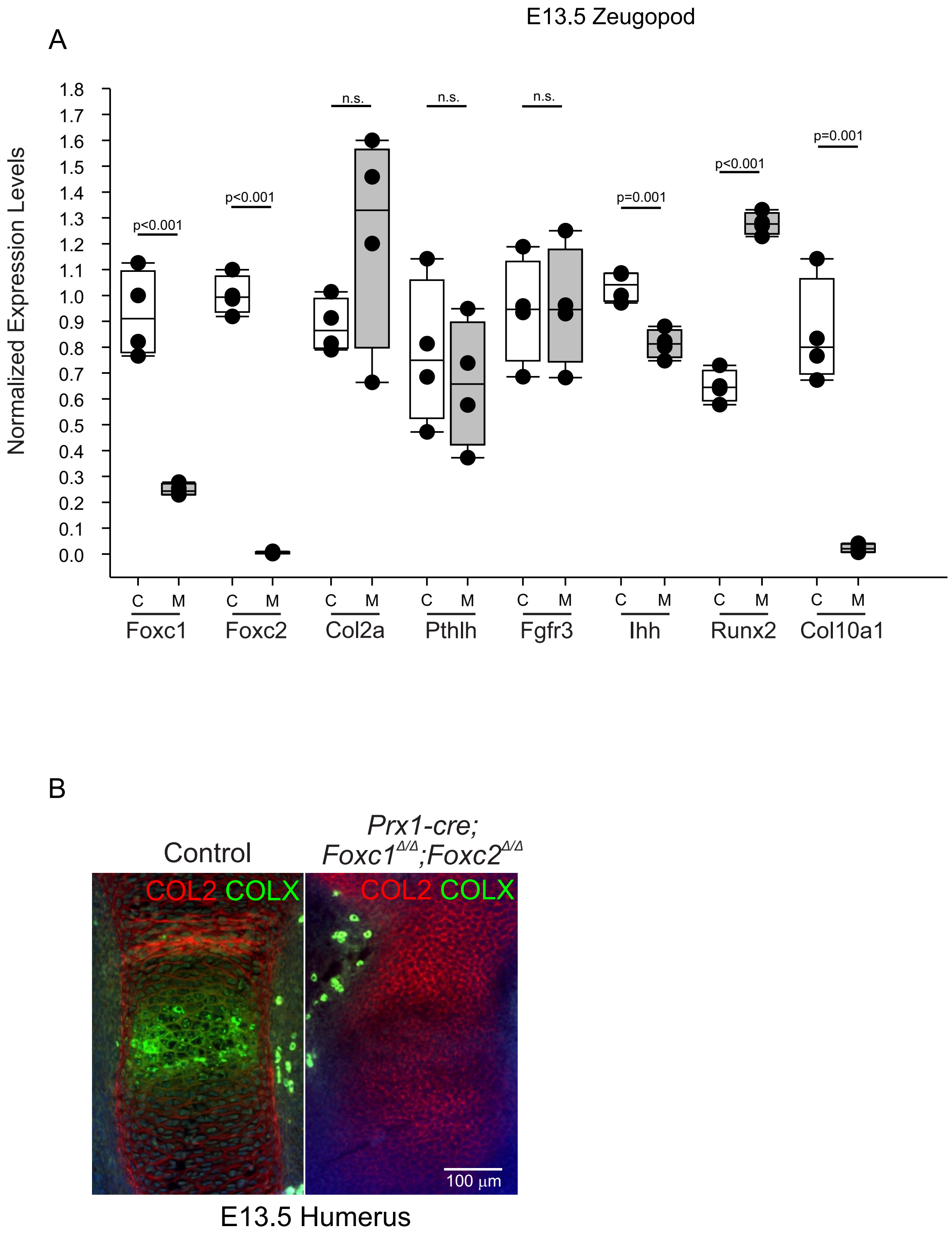

Development • Supplementary information

Fig. S2. Reduced Ihh and Colx mRNA levels in the E13.5 hindlimb zeugopod in *Prx1-cre;Foxc1$^{\Delta/\Delta}$;Foxc2$^{\Delta/\Delta}$* embryos. (A) RNA was isolated from the limbs of four control (C) and four *Prx1-cre;Foxc1$^{\Delta/\Delta}$;Foxc2$^{\Delta/\Delta}$* (M) embryos. Expression levels are normalized to HPRT and 18S RNAs. Statistical analysis was performed by one way ANOVA. (n=4). (B) COLX protein was localized to the HZ of the humerus at E13.5 in control embryos but not in *Prx1-cre;Foxc1$^{\Delta/\Delta}$;Foxc2$^{\Delta/\Delta}$* mutants. Results were comparable between three embryos collected for each genotype

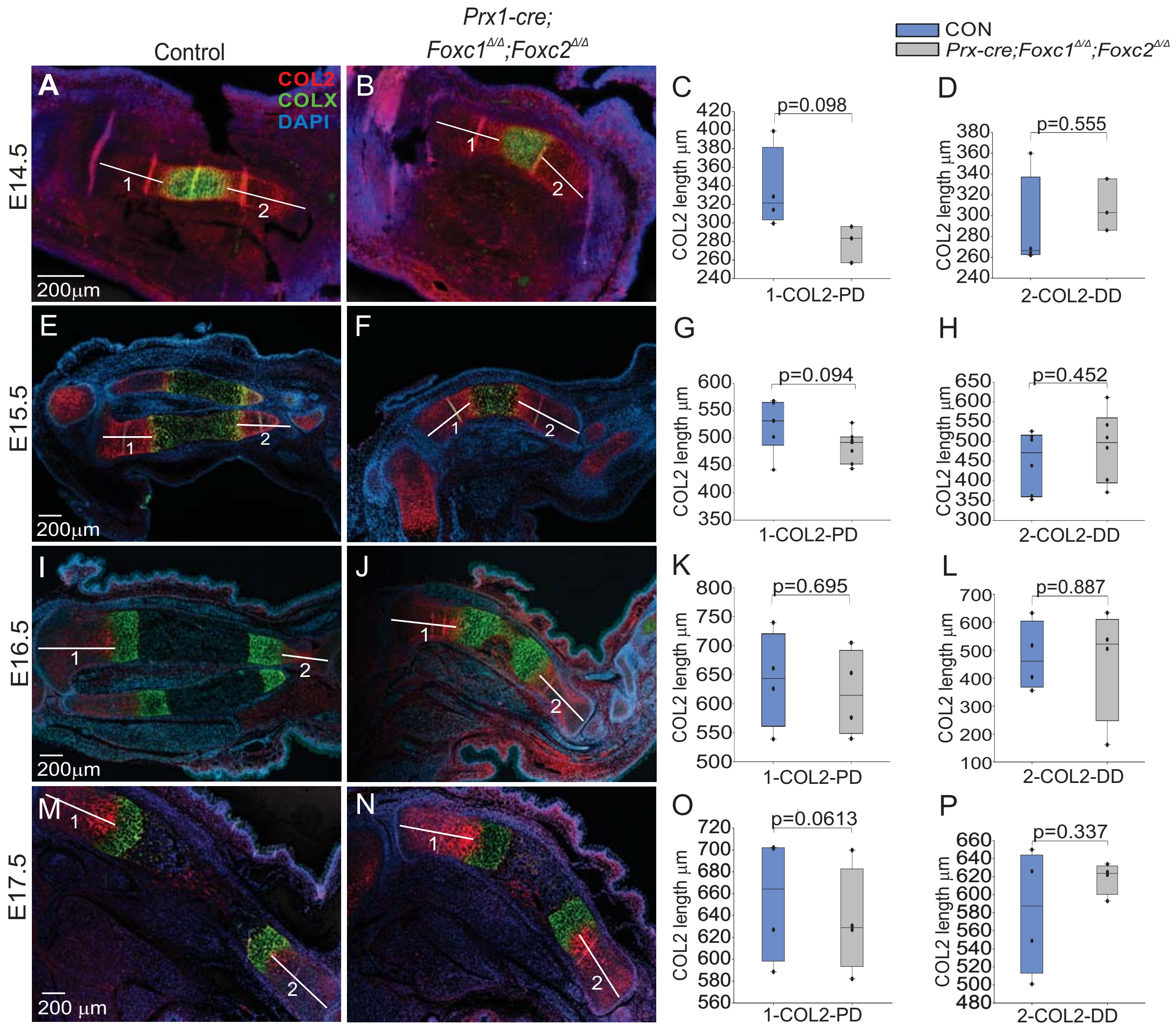

Fig. S3. Length of COL2-expressing chondrocyte zone is unaffected by deletion of *Foxc1* and *Foxc2*.

Comparison of the COL2 (red) and COLX (green) chondrocytes in control and *Prx1-cre;Foxc1*$^{\Delta/\Delta}$*;Foxc2*$^{\Delta/\Delta}$ tibia. The length of the COL2 (red) chondrocytes was measured from the distal and proximal tibia at E14.5 (A-D), E15.5 (E-H), E16.5 (I-L) and E17.5 (M-P). The length of COL2 positive chondrocyte zone did not significantly change. Scale bar 200 μm. Statistical analysis was performed using Student t-test. (n=4).

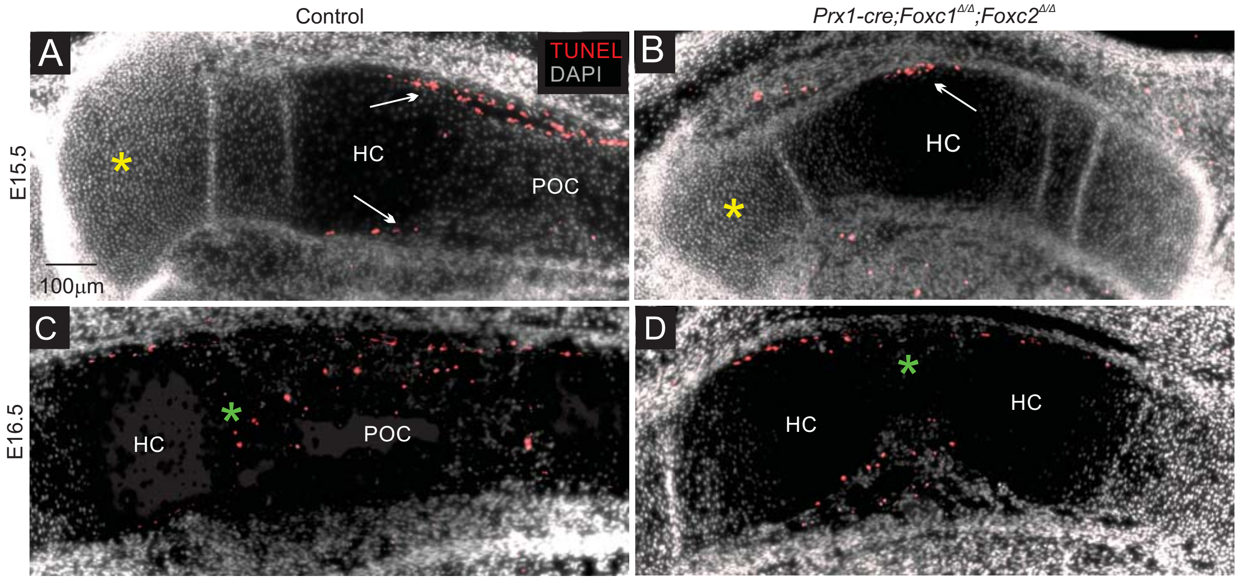

Fig. S4. Expanded hypertrophic chondrocyte zone is not a result of decreased cell death in the growth plate of *Prx1-cre;Foxc1$^{\Delta/\Delta}$;Foxc2$^{\Delta/\Delta}$* limbs.

Cell death was monitored by TUNEL assays. was performed in E15.5 and E16.5 control and *Prx1-cre;Foxc1$^{\Delta/\Delta}$;Foxc2$^{\Delta/\Delta}$* tibia. Distinctive apoptosis activity in a cell population adjacent to the perichondrium and the periosteum (white arrows) was observed at E15.5. However, no cell death was detected in the growth plate chondrocytes in both control and *Prx1-cre* mutant limbs (yellow asterisk) (A, B). At E16.5 control limbs displayed some TUNEL signal in the POC (green asterisk; C) . Reduced TUNEL signal was detected within the smaller POC in the *Prx1-cre;Foxc1$^{\Delta/\Delta}$;Foxc2$^{\Delta/\Delta}$* embryos (green asterisk; D). (n=3)

Development • Supplementary information
