# Supplementary material for: FOXC1 and FOXC2 regulate growth plate chondrocyte maturation towards hypertrophy in the embryonic mouse limb skeleton
Source: Development. 2024 Aug 22;151(16):dev202798. doi: 10.1242/dev.202798 (PMC11361642; doi:10.1242/dev.202798)
Supplement: Supplementary file 1 [file develop-151-202798_review_history.pdf]

Foxc1 and Foxc2 function in growth plate chondrocyte maturation towards hypertrophy and regulate formation of the primary ossification center in mouse embryonic limb skeleton development.

Asra Almubarak, Qiuwan Zhang, Cheng-Hai Zhang, Noor Abdelwahab, Tsutomu Kume, Andrew B Lassar and Fred B Berry
DOI: 10.1242/dev.202798

Editor: Liz Robertson

Review timeline
Original submission: 2 May 2023
Editorial decision: 30 May 2023
Resubmission: 20 February 2024
Editorial decision: 12 March 2024
First revision received: 14 June 2024
Accepted: 1 July 2024

Original submission

First decision letter

MS ID#: DEVELOP/2023/201937

MS TITLE: Foxc1 and Foxc2 function in osteochondral progenitors for the progression through chondrocyte hypertrophy and mineralization of the primary ossification center.

AUTHORS: Asra Almubarak, Qiuwan Zhang, Cheng-Hai Zhang, Tsutomu Kume, Andrew Lassar, and Fred Berry

Dear Dr. Berry,

I have now received all the referees' reports on the above manuscript, and have reached a decision. The referees' comments are appended below, or you can access them online: please go to BenchPress and click on the 'Manuscripts with Decisions' queue in the Author Area.

As you will see, the referees express considerable interest in your work, but have some significant criticisms and recommend a substantial revision of your manuscript before we can consider publication. If you are able to revise the manuscript along the lines suggested, which may involve further experiments, I will be happy receive a revised version of the manuscript. Your revised paper will be re-reviewed by one or more of the original referees, and acceptance of your manuscript will depend on your addressing satisfactorily the reviewers' major concerns. Please also note that Development will normally permit only one round of major revision.

If it would be helpful, you are welcome to contact us to discuss your revision in greater detail. Please send us a point-by-point response indicating your plans for addressing the referee’s comments, and we will look over this and provide further guidance.

Please attend to all of the reviewers' comments and ensure that you clearly highlight all changes made in the revised manuscript. Please avoid using 'Tracked changes' in Word files as these are lost in PDF conversion. I should be grateful if you would also provide a point-by-point response detailing

© 2024. Published by The Company of Biologists under the terms of the Creative Commons Attribution License (https://creativecommons.org/licenses/by/4.0/).

how you have dealt with the points raised by the reviewers in the 'Response to Reviewers' box. If you do not agree with any of their criticisms or suggestions please explain clearly why this is so.

Reviewer 1

Advance summary and potential significance to field

Almubarak, colleagues and collaborators pursued here investigations on the importance of the Foxc1 and Foxc2 genes in skeletogenesis. They focused on limb skeletogenesis, which had not been fully studied in previous studies. They primarily analyzed mouse embryos in which the two genes were inactivated in limb bud skeletogenic mesenchyme using Prx1Cre, and they generated largely descriptive data at tissue, cellular and gene expression levels. The study has numerous valuable findings, but there are also numerous issues that challenge major conclusions reached by the authors.

Comments for the author

Valuable findings and issues with data generation and description
1/ The authors made the interesting finding that Foxc1/2 are more critical for the development of appendicular structures that form later than for those that form earlier (autopod > zeugopod > stylopod, and ulna and fibula > radius and tibia). It is disappointing, however, that they did not investigate the underlying mechanism, that is, how important both genes (or either gene) are to amplify, maintain and specify limb bud mesenchyme over time along the proximal-distal and anterior-posterior axes. This investigation should start with a detailed analysis of where and how strongly each gene is expressed in early limb bud mesenchyme (embryonic stages) and in developing appendicular skeleton structures (fetal stages), along with mesenchymal and cell lineage markers (e.g., Msx1 and Sox9).

2/ The data obtained with Sox9Cre are dispensable as they do not provide any new information. I would recommend removing them from the manuscript.

3/ Figure 2. The authors show valuable data on the expression levels of Foxc1 and Foxc2 in developing limbs, but they are insufficient to convince the readers that Foxc1 and Foxc2 are more expressed in the autopod than zeugopod and stylopod because data are missing to compare the stylopod, zeugopod and autopod at equivalent stages of skeleton development. Indeed, the E14.5 tibia is already more developed than the autopod skeletal structures at E16.5. In fact, the data suggest that Foxc1 and Foxc2 are primarily expressed in perichondrium surrounding immature, early-proliferating chondrocytes in cartilage primordia (before the onset of growth plate formation) and in growth plates, as their expression level looks similar in the perichondrium surrounding the tibia epiphyses and columnar zones and the autopod cartilage primordia. Additional in situs at earlier (E12.5/E13.5 stylopod, zeugopod and autopod elements) and later (E18.5) developmental stages would help generate more convincing data. Moreover, since the fibula and ulna fail to form in mutants, it would be valuable to show whether the two genes are more strongly expressed in the primordia of these elements than in those of the tibia and radius.

4/ Figure 3. In the images shown in the panels A and B, the control sample appears to have more proliferating cells (KI67 positive cells) outside cartilage (in perichondrium and muscle) than the mutant sample, but that both samples have similar proportions of proliferating chondrocytes. Therefore, the conclusion reached by the authors that Foxc1/2 regulate chondrocyte proliferation does not seem appropriate. It is unclear which cell types were included in KI67 positivity counts. Did the authors include all cells shown in their boxes (chondrocytes, perichondrium cells, and soft tissue cells) or only chondrocytes? Moreover, did they count cells in equivalent domains in control and mutant tissues (e.g., only chondrocytes in immature cartilage regions) or did they count all cells in boxes made at the same size regardless of differences in size and maturation stage of control and mutant structures, and regardless of the age of the embryos? Representative images of the tissue regions analyzed should be shown for each developmental stage, not for E14.5 only, and the tissue regions in which cells were counted for KI positivity should be clearly demarcated. Importantly, how do the authors explain that their proliferation assays suggest a drastic reduction

© 2024. Published by The Company of Biologists under the terms of the Creative Commons Attribution License (https://creativecommons.org/licenses/by/4.0/).

in chondrocyte proliferation in mutant tibias, but that their skeletal preparations and histology data show no major difference in length between control and mutant tibias at E15.5 and E18.5?

5/ Figure 4. The conclusion of the authors that Foxc1/2 inactivation “slows the formation of Ihh-expressing pre-hypertrophic chondrocytes” is not supported by the data shown in Figure 4. The data show that E14.5 controls have more Ihh-positive cells than mutants, but this does not prove that the former formed earlier than the latter. Data generated at E12.5 or E13.5 would be necessary to validate or invalidate the authors’ conclusion. It is likely that the mutant elements were delayed in initiating Ihh expression but this could have been a secondary consequence of a delay in forming cartilage primordia rather than a primary consequence of Foxc1/2 inactivation.

6/ Figures 5 and 6. The expanded domain of collagen type 10 seen in mutants around E16.5 is quite certainly a consequence of the overall developmental delay of the tibia rather than a true impairment of hypertrophic chondrocyte turnover. The authors should present their data on a single graph for all time points, with the Y-axis scale starting at 0. They should revise their quantification for the controls at E15.5 because it appears that they included the primary ossification center in their quantification of the hypertrophic zone. This center may contain remnants of cartilage that are positive for collagen type 10, but these remnants should not be counted in the hypertrophic chondrocyte zone. Finally, we would recommend that they include data for E15.0 controls, as these data would likely show that these controls are at the same early stage of endochondral ossification as the mutants are at E16.5, with a relatively enlarged hypertrophic zone compared to that seen at later time points.

7/ Figure 7. The morphological aspect of the E17.5 mutant tibia shown in panel R strongly suggests to us that the region labeled as “POC” is still populated by terminally differentiated hypertrophic chondrocytes rather than being a primary ossification center. The bending of the tibia that is seen to be associated with a wedge of bone collar signifies a possibly impaired attempt of bone-forming cells to invade the cartilage. However, there may not be any specific mutant impairment here, as this phenomenon is often seen transiently in wild-type skeletal elements just prior to endochondral ossification. Better-quality images would help validate this point. Thus, the conclusion of the authors that osteoblast formation and endochondral bone mineralization are impaired in the mutants is not substantiated by the data.

8/ Figures 8 and 9. The data on gene expression are not convincing in the absence of quantification and analysis at successive timepoints. The validity of the conclusions reached by the authors is thus unclear.

Examples of text issues
1/ Title. Data presented in the study do not support the statement that Foxc1 and Foxc2 are necessary in osteochondral progenitors - rather than in downstream cells - for chondrocyte hypertrophy and mineralization of the primary ossification center. Also, no solid data are presented to indicate that Foxc1/2 are needed for chondrocyte hypertrophy and bone mineralization, as such defects are likely secondary rather than primary consequences of the gene loss.

2/ Abstract. (i) Foxc1/2 are only mentioned to be expressed in condensing mesenchyme prior to chondrogenesis, even though the genes are also expressed in some cells later. This incomplete expression pattern description does not explain the findings described in the rest of the abstract and therefore leaves readers unsatisfied, if not confused. (ii) Is Prx1Cre active in lateral plate mesoderm or in lateral plate mesoderm-derived limb bud mesenchyme? (iii) E14.5 is not defined. (iv) The expansion of the COLX domain at E14.5 likely reflects a developmental delay rather than impaired turnover of hypertrophic chondrocytes. (v) “Levels of osteopontin were elevated in the POC…, while expression of Phex was reduced, indicating…”. Are authors referring to the RNA or protein levels of osteopontin and Phex? This should be specified and the word “indicating” should be replaced by “suggesting”.

3/ Introduction. (i) As written, the first paragraph gives the impression that all mesoderm-derived mesenchymal cells give rise to chondrocytes. It is then unclear where perichondrium/periosteum cells and osteoblasts come from. (ii) A written, it appears that all hypertrophic chondrocytes give rise to osteoblasts, which is incorrect. (iii) Sox9 is only said to be expressed in condensing mesenchymal cells and to regulate the expression of the collagen 2 gene. This is incorrect and

© 2024. Published by The Company of Biologists under the terms of the Creative Commons Attribution License (https://creativecommons.org/licenses/by/4.0/).

misleading. (iv) The paragraph introducing the Foxc1 and Foxc2 transcription factors is incomplete and vague. First, the authors should tell us how similar the two proteins are, as this is essential to suggest that they could have additive or redundant functions where co-expressed. Second, when writing that "these factors display overlapping expression in skeletal progenitors of the limbs and vertebrae", the authors should spell out the spatial, temporal and quantitative extent of overlapping expression (for instance, are they expressed at similar levels in most cells and at most developmental stages, or are they co-expressed only in a few cells at a very specific time point, but otherwise expressed differentially?). Third, when saying that mice with inactivation of either Foxc1 or Foxc2 display axial skeletal malformations, it would be relevant to tell us whether these mice had a global or conditional inactivation of the genes, what kind of malformations they had, what the extent of severity of these malformations was, and whether the lack of severe defects in limbs was due to the type of Cre transgene used to inactivate the genes. Fourth, when saying that Col2Cre mutant had "impaired chondrocyte differentiation", it would be relevant to specify what type of impairment was observed.

4/ Final paragraph in the discussion. Authors indicate that they have shown "overlapping roles for Foxc1 and Foxc2 in….". They only analyzed compound mutant mice. They should thus rephrase this sentence. The second sentence regarding roles for the factors at two phases in endochondral ossification should also be revised when solid data are obtained. If new data support specific roles for Foxc1/2 in endochondral ossification, then the authors should at least propose a possible mechanism to explain why these roles are more prominent in distal than proximal skeletal elements.

5/ A discussion in the introduction or discussion on the types of transcription factors that Foxc1 and Foxc2 are, on their known or likely gene targets in skeletogenesis and other biological processes, and on whether the human genes have been associated with genetic or acquired diseases would be welcome.

Examples of minor issues in figures
1/ Mutant labelings should include Prx1Cre, not just Foxc1D/D;Foxc2D/D.
2/ In situ images. Authors should not be afraid of increasing the brightness of images to help visualize signals better. In addition, the sharpness and resolution of many images should be improved.
3/ Figure 1C-F. These data are dispensable.
4/ Figure 1G. Scale bars are missing for the enlarged images at E18.5 and E15.5.
5/ Figure 2E-H. The boxes in panels E and F should be labeled G and H, rather than F and G.
6/ Figure 9. It would be helpful to write OPN, Phex and Foxc1 in the same color as their respective in situ signals.

Reviewer 2

Advance summary and potential significance to field

This manuscript provided data in support of an overlapping role for Foxc1 and Foxc2 transcription factors in mouse limb development. Their findings if substantiated provide new insight in the regulation of chondrocyte hypertrophy in endochondral ossification, regulating entry as well as exit of hypertrophy chondrocytes. This is highly relevant to biologist working in skeletal development as well as associated rare diseases of the skeleton.

Comments for the author

This is a well-written manuscript providing a descriptive assessment for the role of Foxc transcription factors in the limb development. They initially performed a conditional inactivation of Foxc1 and Foxc2 genes in mice using the Sox9-iresCre that showed a difference in its impact on the development of the paraxial and appendicular skeletal structures. Given the mutant embryos were not viable after E12.5, the authors elected to study the impact on limb development using the Prx1-Cre to conditionally inactivate these genes in limb bud mesenchyme.
They reported an overlapping function for Foxc1 and Foxc2 in limb bud mesenchyme regulating endochondral ossification at a specific window in development, affecting mostly distal skeletal

© 2024. Published by The Company of Biologists under the terms of the Creative Commons Attribution License (https://creativecommons.org/licenses/by/4.0/).

structure that included the autopods that are more severe that zeugopods. A systematic assessment using specific molecular markers for endochondral ossification was performed to study the initiation of hypertrophy, from the formation of the primary ossification centre (POC) to the establishment of the growth plate. They concluded that Foxc1 and Foxc2 function at two phases in endochondral ossification: at the differentiation of chondrocyte progenitors to hypertrophy, and later in the remodelling of the hypertrophic cartilage permissive for the formation of the POC and marrow space. The major changes observed is between E14.5-E15.5. While their conclusions in most parts are supported by the data, a critical question is whether the changes are due to a developmental delay rather than a specific impact on the entry and exit of hypertrophic chondrocytes. While this could be challenging, the concern should be addressed experimentally and discussed as a limitation in the interpretation of the data.
Specific comments:
The differences between immunostaining for collagen X and in situ for Col10a1 (Figure 5) is intriguing and puzzling. Do the authors have good statistical data on this finding? Also, to truly showed that there is a difference, it would be important to perform both assessments on the same section (very challenging), or on consecutive sections (this should be attempted) to substantiate the claim. The region of hypertrophic chondrocytes would best illustrated by Col10a1 expression, and not localization as Collagen X can diffuse due to unknown changes in the extracellular matrix environment.
Similarly, the mineralization study in Figure 7 could also be due to developmental delay. It looks like the mutant mice are about one day behind in development. Perhaps the authors could consider showing data for E14.5 as an early developmental reference.
The interpretation on the “entry and exit from chondrocyte hypertrophy” can be strengthened. Given that there is no change in the Collagen II expressing domain, one would interpret that entry is not affected, as this would lead to an expanded proliferative zone. The reduced Ihh expression zone could be due to enhanced maturation process to full hypertrophy, and thus an expanded hypertrophic zone. Further, while apoptosis is one exit route of hypertrophy chondrocytes, this is minimal, and the authors showed no differences. The other is the transition to osteoblasts as demonstrated by multiple labs recently. This could also explain the expanded hypertrophic zone and delayed mineralization.
Minor points:
P14, line 281. Not sure the authors can claim that Foxc1 and Foxc2 are essential. They have a role, and mineralization occurs ablate reduced/delayed.
P15, line 308. Similarly, cannot claim “essential” and “necessary”, they are only part of the equation, and are require for proper remodelling of the hypertrophic zone.
Figure 2. Place some labels to annotate the different bones would be helpful. The panel numbering is a bit confusing with a subpanel G and a main panel G.
Figure 3. Difficult to appreciate there is a reduced number of proliferating cells as there is also an overall reduced size of the cartilage analgen. Perhaps better description of the method of assessment would help, and any normalization involved. Given the data is available, the authors should show representative staining for all the time point assessed.
Figure 5. Consider adding the gene labels in the panels of the mutant mice.
Figure 9. Consider using white text for the panels, as blue can be very hard for many to visualize against a black background.
Figure S2. While in the main text, it is described no change in the level of SOX9 and SOX6 staining, relative to controls, there seem to be an increase in the mutant bones from the pictures shown.

Reviewer 3

Advance summary and potential significance to field

Almubarak et al. present findings here that define the role of transcription factors Foxc1 and Foxc2 specifically in limb skeletal development. In previous work, loss of these factors in chondrocytes alone presented with more severe defects in the axial skeletal elements. As these TFs are expressed in both axial and limb, the authors sought to investigate the role in the limb earlier in development and specifically in the appendicular skeleton using Prx-cre and dual floxxed alleles.

Comments for the author

© 2024. Published by The Company of Biologists under the terms of the Creative Commons Attribution License (https://creativecommons.org/licenses/by/4.0/).

Interestingly, there appear to be two roles for these TFs in both early chondrogenic element formation (~E14.5) and later during the maturation of hypertrophic chondrocytes (~E16.5). Very nice histological evidence of distal cartilage element maturation being delayed (noted in part by lower proteoglycan deposition) was anatomically correlated with the highest levels of these TFs in the WT embryos. This delay is accompanied by reduced expression domains of IHH and associated receptors. However, I did not see a significant reduction in Gli1,2,3 expression in the cKO embryos as reported in the results. I recommend that the authors clarify or quantify this. Nevertheless, it is clear that IHH and PTH signaling are reduced in the cKO POCs, but functioning.

The second interesting phenotype is the delay of hypertrophy induction and persistence of the hypertrophic chondrocytes in the tibia. While the authors do clearly present the cKO results as a delay in chondrocyte exit from the hypertrophic state, I think it would be interesting to pose what may be compensating, albeit 24-48 hours later, in this process, in the discussion. The co-expression of the TFs with Phex in the POC is also a compelling link. Another point I'd like to see added in the discussion is how Foxc1 and Foxc2 might be regulating Phex in chondrocytes, ie is it known to bind enhancers in the region? It is, however, not necessary to provide gene regulatory data for this manuscript in this reviewer's opinion.

Minor comments

Page 9 line 174 callout to figure panel needs correction.

I do not see explicit explanation of the model in Figure 10.

First revision

Author response to reviewers' comments

We thank the reviewers for their time in assessing this work and their comments and helpful suggestions of this research study. A common issue that was raised from all reviewers was whether the expanded hypertrophic chondrocyte zone and smaller primary ossification center that forms was a consequence of developmental delays from deleting Foxc1 and Foxc2 in limb bud mesenchymal cells. Although our data do support a role for Foxc1 and Foxc2 in regulating chondrogenesis in forming limb leading to delays in progression through chondrocyte hypertrophy, we wanted to test whether Foxc1 and Foxc2 did function in hypertrophic chondrocytes to regulate later stages of endochondral bone formation. To do this we created a ColX-cre conditional Foxc1;Foxc2 mutant line which targets hypertrophic chondrocytes.
Embryos from his mouse model did recapitulate many of the phenotypes we observed in the hypertrophic chondrocytes and in the forming primary ossification center of the Prx1-cre condition mutant. Given that there was not a complete overlap in the bone phenotype suggests a role for Foxc1 and Foxc2 outside of hypertrophic chondrocytes. We did provide data generated from this new model (Figure 7 and starting from line 241) to support our findings obtained with the Prx-cre conditional mutant. We recognize a more comprehensive analysis of the Colx-cre model is required and felt it would stand as its own independent analysis in a future study.

We have addressed the remainder of the reviewers' comments and concerns to the best of our abilities and resources in this revised manuscript. Our modifications are documented below in normal typeface.

Reviewer 1 Advance Summary and Potential Significance to Field:
Almubarak, colleagues and collaborators pursued here investigations on the importance of the Foxc1 and Foxc2 genes in skeletogenesis. They focused on limb skeletogenesis, which had not been fully studied in previous studies. They primarily analyzed mouse embryos in which the two genes were inactivated in limb bud skeletogenic mesenchyme using Prx1Cre, and they generated largely descriptive data at tissue, cellular and gene expression levels. The study has numerous valuable findings, but there are also numerous issues that challenge major conclusions reached by the authors.

Reviewer 1 Comments for the Author:
Valuable findings and issues with data generation and description

© 2024. Published by The Company of Biologists under the terms of the Creative Commons Attribution License (https://creativecommons.org/licenses/by/4.0/).

1/ The authors made the interesting finding that Foxc1/2 are more critical for the development of appendicular structures that form later than for those that form earlier (autopod > zeugopod > stylopod, and ulna and fibula > radius and tibia). It is disappointing, however, that they did not investigate the underlying mechanism, that is, how important both genes (or either gene) are to amplify, maintain and specify limb bud mesenchyme over time along the proximal-distal and anterior-posterior axes. This investigation should start with a detailed analysis of where and how strongly each gene is expressed in early limb bud mesenchyme (embryonic stages) and in developing appendicular skeleton structures (fetal stages), along with mesenchymal and cell lineage markers (e.g., Msx1 and Sox9).

We thank the reviewer for this suggestion. In a previous study (Almburak et al 2021; JBC DOI: 10.1016/j.jbc.2021.101020) we compared Foxc1 and Foxc2 expression in the limb at E12.5 and E13.5 but did not find differences in expression intensities between proximal and distal or anterior posterior limb bone structures at E12.5. We did observe that Foxc1 and Foxc2 expression was restricted to the femur perichondrium by E13.5 and remained expressed throughout the zeugopod and autopod primordia. We have included these findings in the interpretation of our results and the manuscript text has been amended (line 60, page 4; line 351 page 17).

2/ The data obtained with Sox9Cre are dispensable as they do not provide any new information. I would recommend removing them from the manuscript.

Although we understand why the reviewer wishes us to remove this data, we do feel it's important to document these findings in the literature. Furthermore these results aid in our interpretation of a role for Foxc1/c2 in the onset of early chondrogenesis differentiation events. Nonetheless, we have shortened the description of these results in the text.

3/ Figure 2. The authors show valuable data on the expression levels of Foxc1 and Foxc2 in developing limbs, but they are insufficient to convince the readers that Foxc1 and Foxc2 are more expressed in the autopod than zeugopod and stylopod because data are missing to compare the stylopod, zeugopod and autopod at equivalent stages of skeleton development. Indeed, the E14.5 tibia is already more developed than the autopod skeletal structures at E16.5. In fact, the data suggest that Foxc1 and Foxc2 are primarily expressed in perichondrium surrounding immature, early-proliferating chondrocytes in cartilage primordia (before the onset of growth plate formation) and in growth plates, as their expression level looks similar in the perichondrium surrounding the tibia epiphyses and columnar zones and the autopod cartilage primordia. Additional in situs at earlier (E12.5/E13.5 stylopod, zeugopod and autopod elements) and later (E18.5) developmental stages would help generate more convincing data. Moreover, since the fibula and ulna fail to form in mutants, it would be valuable to show whether the two genes are more strongly expressed in the primordia of these elements than in those of the tibia and radius.

We have previously performed in situ hybridization on E12.5 and E 13.5 hindlimbs for Foxc1 and Foxc2 (Almburak et al 2021; JBC DOI: 10.1016/j.jbc.2021.101020). As early as E13.5 we observed Foxc1 and Foxc2 expression being restricted to the femur perichondrium while expression in distal structures is prominent throughout the limb anlage. We have amended the text (line 60, page 4; line 351 page 17) to emphasize the findings of this research with respect to our interpretation of the result of our current study.

4/ Figure 3. In the images shown in the panels A and B, the control sample appears to have more proliferating cells (KI67 positive cells) outside cartilage (in perichondrium and muscle) than the mutant sample, but that both samples have similar proportions of proliferating chondrocytes. Therefore, the conclusion reached by the authors that Foxc1/2 regulate chondrocyte proliferation does not seem appropriate. It is unclear which cell types were included in KI67 positivity counts. Did the authors include all cells shown in their boxes (chondrocytes, perichondrium cells, and soft tissue cells) or only chondrocytes? Moreover, did they count cells in equivalent domains in control and mutant tissues (e.g., only chondrocytes in immature cartilage regions) or did they count all cells in boxes made at the same size regardless of differences in size and maturation

© 2024. Published by The Company of Biologists under the terms of the Creative Commons Attribution License (https://creativecommons.org/licenses/by/4.0/).

stage of control and mutant structures, and regardless of the age of the embryos? Representative images of the tissue regions analyzed should be shown for each developmental stage, not for E14.5 only, and the tissue regions in which cells were counted for KI positivity should be clearly demarcated. Importantly, how do the authors explain that their proliferation assays suggest a drastic reduction in chondrocyte proliferation in mutant tibias, but that their skeletal *preparations and histology data show no major difference in length between control and mutant tibias at* E15.5 and E18.5?

In Figure 1 we added additional data where we measured the length of the femur and tibia in our skeletal preparation images. We also ensured images presented in this figure were of the same magnification. Our results demonstrate the femur and tibia are shorter in the Prx1-cre Foxc1/c2 conditional mutants compared to controls, although malformations to the femur are not evident. For the Ki 67 assays, we asked whether cell proliferation changes in the developing limb could account for the size differences we observed in long bones of the Prx1-cre mutants. We measured the percentage of KI67- positive cells in the areas outlined. We strived to identify cells in the growth plate but we recognize that additional cells (non-chondrocytes) could be included in this analysis. Additional images were added to Figure 3 representing all the time points analyzed.
We provided additional information on our analysis in the Material and Methods Sections (line 521, page 25)

5/ Figure 4. The conclusion of the authors that Foxc1/2 inactivation "slows the formation of Ihh-expressing pre-hypertrophic chondrocytes" is not supported by the data shown in Figure 4. The data show that E14.5 controls have more Ihh-positive cells than mutants, but this does not prove that the former formed earlier than the latter. Data generated at E12.5 or E13.5 would be necessary to validate or invalidate the authors' conclusion. It is likely that the mutant elements were delayed in initiating Ihh expression but this could have been a secondary consequence of a delay in forming cartilage primordia rather than a primary consequence of Foxc1/2 inactivation.

To address this concern, we isolated RNA from the hindlimb zeugopod at E13.5 from four control and four *Prx1-cre;Foxc1*$^{\Delta/\Delta}$*;Foxc2*$^{\Delta/\Delta}$ embryos and measured Ihh, and Col10a expression. We found that Ihh expression was reduced by 1.5x in the mutants, whereas Col10a mRNA levels were reduced by over 60 fold in the Prx1-cre mutants. We also measured Foxc1 and Foxc2 expression at this time point and found that while Foxc2 expression was undetectable in the mutants, we could detect some Foxc1 expression in the mutants (~25 % of controls). Thus residual Foxc1 may be present when stylopod bone elements form and lessen phenotype severity. These results were added to manuscript as Figure S2 and discuss in the text on line 181, page 9 and line 357, page 17.

6/ Figures 5 and 6. The expanded domain of collagen type 10 seen in mutants around E16.5 is quite certainly a consequence of the overall developmental delay of the tibia rather than a true impairment of hypertrophic chondrocyte turnover. The authors should present their data on a single graph for all time points, with the Y-axis scale starting at 0. They should revise their quantification for the controls at E15.5 because it appears that they included the primary ossification center in their quantification of the hypertrophic zone. This center may contain remnants of cartilage that are positive for collagen type 10, but these remnants should not be counted in the hypertrophic chondrocyte zone. Finally, we would recommend that they include data for E15.0 controls, as these data would likely show that these controls are at the same early stage of endochondral ossification as the mutants are at E16.5, with a relatively enlarged hypertrophic zone compared to that seen at later time points.

We do agree that loss of Foxc1 and Foxc2 in Prx1-cre expressing cells is delaying limb skeletal development. In order to test whether delay alone accounts for expansion of the hypertrophic zone, we generated a Colx-cre Foxc1;Foxc2 condition mutant model to determine if Foxc1 and Foxc2 function in hypertrophic chondrocytes. *ColX-cre;Foxc1*$^{\Delta/\Delta}$*;Foxc2*$^{\Delta/\Delta}$ embryos were smaller than their control littermates but had no overt skeletal malformations. We observed expansion of COLX protein localization in the HC zone and a shortened primary ossification center in the Colx-cre conditional mutants that was similar to the Prx1-cre mutants. These findings do support a

© 2024. Published by The Company of Biologists under the terms of the Creative Commons Attribution License (https://creativecommons.org/licenses/by/4.0/).

role for Foxc1 and Foxc2 in HC to control their differentiation and indicate that developmental delay is not the sole reason for HC zone expansion. These new data have been added as Figure 7 and included in the manuscript text (line 241. page 12). We recognize that our analysis of Colx-cre mutants is limited given the time constraints to provide manuscript revision. We did modify the graphs in Figure 6 to incorporate the reviewer's suggestions.
However we did not change the measurement of the HC zone at E15.5 in Figure 6. Instead we changed the label to reflect what was being measured, the length of Colx-expressing cells. We wanted this figure to capture what was observed in our immunofluorescence experiments

7/ Figure 7. The morphological aspect of the E17.5 mutant tibia shown in panel R strongly suggests to us that the region labeled as "POC" is still populated by terminally differentiated hypertrophic chondrocytes rather than being a primary ossification center. The bending of the tibia that is seen to be associated with a wedge of bone collar signifies a possibly impaired attempt of bone-forming cells to invade the cartilage. However, there may not be any specific mutant impairment here, as this phenomenon is often seen transiently in wild-type skeletal elements just prior to endochondral ossification. Better-quality images would help validate this point. Thus, the conclusion of the authors that osteoblast formation and endochondral bone mineralization are impaired in the mutants is not substantiated by the data.

We agree with the reviewer's assertion the POC has likely yet to form in the E17.5 mutant. We referred to it as a POC to be consistent in our terminology when compared to the control embryo. We took reviewer's recommendation and redesigned this figure using higher resolution images, we also included quantification of these data to demonstrate that fewer OSX-positive osteoblast progenitors are populating the region that will form the primary ossification center in the Prx1-cre mutants.

Examples of text issues

1/ Title. Data presented in the study do not support the statement that Foxc1 and Foxc2 are necessary in osteochondral progenitors - rather than in downstream cells - for chondrocyte hypertrophy and mineralization of the primary ossification center. Also, no solid data are presented to indicate that Foxc1/2 are needed for chondrocyte hypertrophy and bone mineralization, as such defects are likely secondary rather than primary consequences of the gene loss.

2/ Abstract. (i) Foxc1/2 are only mentioned to be expressed in condensing mesenchyme prior to chondrogenesis, even though the genes are also expressed in some cells later. This incomplete expression pattern description does not explain the findings described in the rest of the abstract and therefore leaves readers unsatisfied, if not confused. (ii) Is Prx1Cre active in lateral plate mesoderm or in lateral plate mesoderm-derived limb bud mesenchyme? (iii) E14.5 is not defined. (iv) The expansion of the COLX domain at E14.5 likely reflects a developmental delay rather than impaired turnover of hypertrophic chondrocytes. (v) "Levels of osteopontin were elevated in the POC…, while expression of Phex was reduced, indicating…". Are authors referring to the RNA or protein levels of osteopontin and Phex? This should be specified and the word "indicating" should be replaced by "suggesting".

The abstract has been re-written to include the suggestions and address the issues raised by the reviewer.

3/ Introduction. (i) As written, the first paragraph gives the impression that all mesoderm-derived mesenchymal cells give rise to chondrocytes. It is then unclear where perichondrium/periosteum cells and osteoblasts come from. (ii) A written, it appears that all hypertrophic chondrocytes give rise to osteoblasts, which is incorrect. (iii) Sox9 is only said to be expressed in condensing mesenchymal cells *and to regulate the expression of the collagen 2 gene. This is incorrect and misleading. (iv) The paragraph* introducing the Foxc1 and Foxc2 transcription factors is incomplete and vague. First, the authors should tell us how similar the two proteins are, as this is

© 2024. Published by The Company of Biologists under the terms of the Creative Commons Attribution License (https://creativecommons.org/licenses/by/4.0/).

essential to suggest that they could have additive or redundant functions where co-expressed. Second, when writing that “these factors display overlapping expression in skeletal progenitors of the limbs and vertebrae”, the authors should spell out the spatial, temporal and quantitative extent of overlapping expression (for instance, are they expressed at similar levels in most cells and at most developmental stages, or are they co-expressed only in a few cells at a very specific time point, but otherwise expressed differentially?). Third, when saying that mice with inactivation of either Foxc1 or Foxc2 display axial skeletal malformations, it would be relevant to tell us whether these mice had a global or conditional inactivation of the genes, what kind of malformations they had, what the extent of severity of these malformations was, and whether the lack of severe defects in limbs was due to the type of Cre transgene used to inactivate the genes. Fourth, when saying that Col2Cre mutant had “impaired chondrocyte differentiation”, it would be relevant to specify what type of impairment was observed.

The introduction has been rewritten to correct the mistakes and to address comments listed above. We included further information regarding our knowledge of Foxc1 and Foxc2 in the development skeletal system. Expression patterns in the developing skeletal have been added as well; we have indicated that previous Foxc1 and Foxc2 mutants were global (germline) mutants. We also describe further the chondrocytes differentiation phenotypes we observed in on Col2-cre mutants.

4/ Final paragraph in the discussion. Authors indicate that they have shown “overlapping roles for Foxc1 and Foxc2 in….”. They only analyzed compound mutant mice. They should thus rephrase this sentence. The second sentence regarding roles for the factors at two phases in endochondral ossification should also be revised when solid data are obtained. If new data support specific roles for Foxc1/2 in endochondral ossification, then the authors should at least propose a possible mechanism to explain why these roles are more prominent in distal than proximal skeletal elements

We reworded this sentence to remove discussion of “overlapping roles for Foxc1 and Foxc2” We have indicated our study of only the double mutants as a limitation to th this study and we are unable to asses the individual roles for Foxc1 and Foxc2 in formation of the skeleton..

5/ A discussion in the introduction or discussion on the types of transcription factors that Foxc1 and Foxc2 are, on their known or likely gene targets in skeletogenesis and other biological processes, and on whether the human genes have been associated with genetic or acquired diseases would be welcome.

We have added additional data as to the molecular properties of Foxc1 and Foxc2, roles in human disease as well as elaborate further on expression patterns in the skeletal system and phenotypes observed in global Foxc1 or Foxc2 mutants.

Examples of minor issues in figures

1/ Mutant labelings should include Prx1Cre, not just Foxc1D/D;Foxc2D/D.

2/ In situ images. Authors should not be afraid of increasing the brightness of images to help visualize signals better. In addition, the sharpness and resolution of many images should be improved.

3/ Figure 1C-F. These data are dispensable.

4/ Figure 1G. Scale bars are missing for the enlarged images at E18.5 and E15.5.

5/ Figure 2E-H. The boxes in panels E and F should be labeled G and H, rather than F and G.

6/ Figure 9. It would be helpful to write OPN, Phex and Foxc1 in the same color as their respective in situ signals.

We have corrected all the above figure changes

© 2024. Published by The Company of Biologists under the terms of the Creative Commons Attribution License (https://creativecommons.org/licenses/by/4.0/).

***** *Reviewer 2 Advance Summary and Potential Significance to Field:*
This manuscript provided data in support of an overlapping role for Foxc1 and Foxc2 transcription factors in mouse limb development. Their findings if substantiated provide new insight in the regulation of chondrocyte hypertrophy in endochondral ossification, regulating entry as well as exit of hypertrophy chondrocytes. This is highly relevant to biologist working in skeletal development as well as associated rare diseases of the skeleton.

Reviewer 2 Comments for the Author:
This is a well-written manuscript providing a descriptive assessment for the role of Foxc transcription factors in the limb development. They initially performed a conditional inactivation of Foxc1 and Foxc2 genes in mice using the Sox9-iresCre that showed a difference in its impact on the development of the paraxial and appendicular skeletal structures. Given the mutant embryos were not viable after E12.5, the authors elected to study the impact on limb development using the Prx1-Cre to conditionally inactivate these genes in limb bud mesenchyme.

They reported an overlapping function for Foxc1 and Foxc2 in limb bud mesenchyme regulating endochondral ossification at a specific window in development, affecting mostly distal skeletal structure that included the autopods that are more severe that zeugopods. A systematic assessment using specific molecular markers for endochondral ossification was performed to study the initiation of hypertrophy, from the formation of the primary ossification centre (POC) to the establishment of the growth plate. They concluded that Foxc1 and Foxc2 function at two phases in endochondral ossification: at the differentiation of chondrocyte progenitors *to hypertrophy, and later in the remodelling of the hypertrophic cartilage permissive for the formation of the* POC and marrow space. The major changes observed is between E14.5-E15.5. While their conclusions in most parts are supported by the data, a critical question is whether the changes are due to a developmental delay rather than a specific impact on the entry and exit of hypertrophic chondrocytes. While this could be challenging, the concern should be addressed experimentally and discussed as a limitation in the interpretation of the data.

We were able to address this issue experimentally by deleting Foxc1 and Foxc2 in hypertrophic chondrocytes using the Colx-cre mouse. These Foxc1/Foxc2 conditional mutants were smaller than the control counterparts, but did not display any overt skeletal malformations. The hypertrophic chondrocyte zone was enlarged in the Colx- cre mutants while the primary ossification center was smaller. Overall the Colx-cre mutants recapitulate many of the hypertrophic chondrocyte phenotypes observed in the prx-cre mutant. These finding suggest that developmental delay was not the sole explanation for the expanded COLX-postive hypertrophic zone observed in Prx1-cre Foxc1 Foxc2 mutants and that Foxc genes may function in hypertrophic chondrocytes to control their differentiation.

Specific comments:

The differences between immunostaining for collagen X and in situ for Col10a1 (Figure 5) is intriguing and puzzling. Do the authors have good statistical data on this finding? Also, to truly showed that there is a difference, it would be important to perform both assessments on the same section (very challenging), or on consecutive sections (this should be attempted) to substantiate the claim. The region of hypertrophic chondrocytes would best illustrated by Col10a1 expression, and not localization as Collagen X can diffuse due to unknown changes in the extracellular matrix environment.

Thank you for this suggestion. We were able to perform COLX immunofluorescence following ColX in situ hybridization. There were inconsistencies between the length of the Colx mRNA and IF signal in our mutants. In some sections the length of Colx mRNA signal was shorter than the IF signal and in others it wasn't. The length of the Colx mRNA or protein signal was always longer in the mutant compared to the control. Because of the differences seen within the mutant sample group we could not conclude that the Colx mRNA signal was unchanged compared to the protein

© 2024. Published by The Company of Biologists under the terms of the Creative Commons Attribution License (https://creativecommons.org/licenses/by/4.0/).

signal and as such this panel was removed from figure 5.

Similarly, the mineralization study in Figure 7 could also be due to developmental delay. It looks like the mutant mice are about one day behind in development. Perhaps the authors could consider showing data for E14.5 as an early developmental reference.

Although this is a good suggestion to test, we felt that the data from Colx-cre support the role for FOXC1 and FOXC2 in hypertrophic chondrocytes in regulation POC formation and the phenotype we observe in the POC of Prx1-cre mice is not necessarily only from developmental delay.

The interpretation on the "entry and exit from chondrocyte hypertrophy" can be strengthened. Given that there is no change in the Collagen II expressing domain, one would interpret that entry is not affected, as this would lead to an expanded proliferative zone. The reduced Ihh expression zone could be due to enhanced maturation process to full hypertrophy, and thus an expanded hypertrophic zone. Further, while apoptosis is one exit route of hypertrophy chondrocytes, this is minimal, and the authors showed no differences. The other is the transition to osteoblasts as demonstrated by multiple labs recently. This could also explain the expanded hypertrophic zone and delayed mineralization.

We agree in the assessment of the overall progression through chondrogensis may be slowed in *Prx1-cre;Foxc1*$^{\Delta/\Delta}$*;Foxc2*$^{\Delta/\Delta}$ which leads to fewer cells entering hypertrophy. We analyzed expression of Ihh and Colx at E13.5 by RT-PCR to assess whether differences in chondrocyte hypertrophy could be assessed (Figure S2). We found that Ihh was reduced by 1.5 x while Colx expression was nearly undetectable (60X reduced) in the Prx1-cre embryos. We interpret this finding to support this notion that chondrogenesis is slowed in the *Prx1-cre;Foxc1*$^{\Delta/\Delta}$*;Foxc2*$^{\Delta/\Delta}$ embryos leading to lower Colx expression rather than an accelerated entry into hypertrophic.

Minor points:

P14, line 281. Not sure the authors can claim that Foxc1 and Foxc2 are essential. They have a role, and mineralization occurs ablate reduced/delayed.

P15, line 308. Similarly, cannot claim "essential" and "necessary", they are only part of the equation, and are require for proper remodelling of the hypertrophic zone.

The above two sections have been rewritten to reflect these changes.

Figure 2. Place some labels to annotate the different bones would be helpful. The panel numbering is a bit confusing with a subpanel G and a main panel G.

We have labeled this figure in accordance with these suggestions

Figure 3. Difficult to appreciate there is a reduced number of proliferating cells as there is also an overall reduced size of the cartilage analgen. Perhaps better description of the method of assessment would help, and any normalization involved. Given the data is available, the authors should show representative staining for all the time point assessed.

We added micrographs for all time points assessed and indicated the regions quantified on each figure. Additional experimental details on this assay were added to the Material and Methods section.

Figure 5. Consider adding the gene labels in the panels of the mutant mice.

These labels have been added

Figure 9. Consider using white text for the panels, as blue can be very hard for many to visualize against a black background.

These modifications have been added.

© 2024. Published by The Company of Biologists under the terms of the Creative Commons Attribution License (https://creativecommons.org/licenses/by/4.0/).

Figure S2. While in the main text, it is described no change in the level of SOX9 and SOX6 staining, relative to controls, there seem to be an increase in the mutant bones from the pictures shown.

This figure has been removed as well as the description in the text that refers to it in order to meet space restrictions. The expression pattern for SOX6 and SOX9 do appear to be elevated in this represented image, but that does not reflect our observations in other experiments. We visualized Sox6 and Sox9 levels as a read out of early stages of chondrogenesis and if the initial chondrocyte formation was disrupted in the mutants, it would be reflected by a reduced levels of Sox9 or Sox6

****** Reviewer 3 Advance Summary and Potential Significance to Field:*
Almubarak et al. present findings here that define the role of transcription factors Foxc1 and Foxc2 specifically in limb skeletal development. In previous work, loss of these factors in chondrocytes alone presented with more severe defects in the axial skeletal elements. As these TFs are expressed in both axial and limb, the authors sought to investigate the role in the limb earlier in development and specifically in the appendicular skeleton using Prx-cre and dual floxxed alleles.

Reviewer 3 Comments for the Author:
Interestingly, there appear to be two roles for these TFs in both early chondrogenic element formation (~E14.5) and later during the maturation of hypertrophic chondrocytes (~E16.5). Very nice histological evidence of distal cartilage element maturation being delayed (noted in part by lower proteoglycan deposition) was anatomically correlated with the highest levels of these TFs in the WT embryos. This delay is accompanied by reduced expression domains of IHH and associated receptors. However, I did not see a significant reduction in Gli1,2,3 expression in the cKO embryos as reported in the results. I recommend that the authors clarify or quantify this.

Quantification of Gli1,2,3 did not reveal any reproducible changes amongst all sections analyzed. We therefore removed this interpretation from the text .

Nevertheless, it is clear that IHH and PTH signaling are reduced in the cKO POCs, but functioning.The second interesting phenotype is the delay of hypertrophy induction and persistence of the hypertrophic chondrocytes in the tibia. While the authors do clearly present the cKO results as a delay in chondrocyte exit from the hypertrophic state, I think it would be interesting to pose what may be
compensating, albeit 24-48 hours later, in this process, in the discussion.

FOXA factors share a conserved DNA binding domain to FOXC proteins. Given that FOXAs can also regulate formation of hypertrophic chondrocytes they are an attractive candidate to compensate for FOXC factors. We did add this explanation to the text (line 400, page 17)

The co-expression of the TFs with Phex in the POC is also a compelling link. Another point I'd like to see added in the discussion is how Foxc1and Foxc2 might be regulating Phex in chondrocytes, ie is it known to bind enhancers in the region? It is, however, not necessary to provide gene regulatory data for this manuscript in this reviewer's opinion.

We do propose that FOXCs proteins may bind to Phex regulatory elements and control its expression. Although this has yet to be experimentally tested, we are currently pursuing this possibility. We have added an explanation that Foxc1 and Foxc2 could directly regulate phex expression in the discussion (line 433, page 20)

Minor comments

Page 9 line 174 callout to figure panel needs correction.

This has been corrected (page 8, line 164)

© 2024. Published by The Company of Biologists under the terms of the Creative Commons Attribution License (https://creativecommons.org/licenses/by/4.0/).

I do not see explicit explanation of the model in Figure 10.

This has been corrected (now Fig 11; page 19 line 393)

Resubmission

First decision letter

MS ID#: DEVELOP/2024/202798

MS TITLE: Foxc1 and Foxc2 function in osteochondral progenitors for the progression through chondrocyte hypertrophy and formation of the primary ossification center.

AUTHORS: Asra Almubarak, Qiuwan Zhang, Cheng-Hai Zhang, Noor Abdelwahab, Tsutomu Kume, Andrew B Lassar, and Fred B Berry

I have now received all the referees reports on the above manuscript, and have reached a decision. The referees' comments are appended below, or you can access them online: please go to BenchPress and click on the 'Manuscripts with Decisions' queue in the Author Area.

The overall evaluation is positive and we would like to publish a revised manuscript in Development, provided that the referees' comments can be satisfactorily addressed. Please attend to all of the reviewers' comments in your revised manuscript and detail them in your point-by-point response. If you do not agree with any of their criticisms or suggestions explain clearly why this is so. If it would be helpful, you are welcome to contact us to discuss your revision in greater detail. Please send us a point-by-point response indicating your plans for addressing the referees' comments, and we will look over this and provide further guidance.

Reviewer 1

Advance summary and potential significance to field

This study was conducted to expand knowledge of the roles of the transcription factors Foxc1 and Foxc2 in the development of appendicular skeletal structures in mouse embryos. It focuses on growth plate chondrocytes and suggests that the two factors act redundantly initially to promote the onset of chondrocyte hypertrophy and subsequently to promote the turnover of these cells at the osteochondral junction. The data are primarily descriptive and currently insufficient or not fully accurately described to fully support the main conclusion. This is unfortunate, as this conclusion, if true, would represent a major advance in current knowledge of the molecular mechanisms involved in the regulation of growth plate chondrocyte differentiation and endochondral ossification.

Comments for the author

Almubarak and collaborators have partially improved their study and manuscript in response to the reviewers' recommendations. Several major issues, however, remain to be addressed to convincingly support the new messages of the study. Many minor ones remain to be attended to too.

Scientific issues
1/ The description of the spatial and temporal expression pattern of Foxc1 and Foxc2 in developing appendicular skeletal elements remains suboptimal. Accurately documenting and describing this expression pattern is important to properly interpret the phenotype of embryos lacking both genes. This issue is seen for instance in the lines 143-145. "… Foxc1 and Foxc2 mRNA signals were prominent in the perichondrium and growth plate, including the hypertrophic chondrocytes…". The

© 2024. Published by The Company of Biologists under the terms of the Creative Commons Attribution License (https://creativecommons.org/licenses/by/4.0/).

actual data show that the expression of two genes is prominent in the perichondrium of cartilage primordia (tarsals, metatarsals, and tibial epiphyses); that it progressively declines in the perichondrium as growth plate chondrocytes mature from the proliferative to the hypertrophic stage (tibial metaphyses); and that it is weak in hypertrophic chondrocytes. Given that the expression of the two genes declines as skeletal cells mature, the stronger expression of the genes in distal compared to proximal elements may reflect the fact that distal elements develop later than proximal elements rather than reflect a true patterning difference between proximal and distal limb structures. Reviewers asked for additional data to address more definitively the question of the expression change between distal and proximal structures, but this was not done in the revised manuscript. It is recommended that this point be addressed more convincingly to help explain the apparent proximo-distal defect of mutant limbs, or, at the minimum, that data be accurately described and interpreted. In addition, it is unclear in the data presented in Figure 2 if hypertrophic chondrocytes are positive for Foxc1 and Foxc2 expression at both E14.5 and E16.5, or only at E14.5. It is recommended that the authors show a close-up image at both stages and that they increase the brightness of their images to address this point. It is important to address this second point because a main message that the authors propose for their study is that mutant growth plate chondrocytes are initially delayed in undergoing hypertrophy, but overcome this defect subsequently, and that this delay is due to cell-autonomous roles of Foxc1 and Foxc2 in maturing chondrocytes. Also note that the panels of Figure 2 are not indicated properly in the main text.

2/ Lines 180-187. As indicated above, a key message of the study proposed by the authors is that the loss of Foxc1 and Foxc2 results in a delay of mutant prehypertrophic chondrocytes to progress to hypertrophy.
The expression data for cell differentiation markers, however, do not support this message since Ihh (prehypertrophic marker) has its expression level reduced rather than having its expression domain increased, as it would be if cells were remaining longer than normal at this stage, and since no data are presented to show whether the early stages of cartilage development (before prehypertrophy) proceed in a proper and timely manner and whether the very first hypertrophic chondrocytes form later in mutant compared to control skeletal elements. Of note, the word “delay” (rather than impairment) should be used here and at other places in the study only if evidence is provided that a feature initially occurs later than expected but is resolved at a later time.

3/ Lines 188-190. Note that the incomplete recombination of Foxc1 in limbs may reflect the fact that Foxc1 may be expressed in cells that are not targeted by Prx1Cre (such as muscle cells) rather than the proposed explanation that Prx1Cre is not efficiently recombining the gene.

4/ Lines 200-202. It is unexpected that Runx2 expression is not detected in control hypertrophic chondrocytes. It appears to be clearly upregulated in mutant hypertrophic chondrocytes, as described by the authors. However, this upregulation does not seem to be specific for hypertrophic chondrocytes since signals for Runx2 are stronger in several other cells too, including perichondrium/periosteum cells, and proliferating and prehypertrophic chondrocytes. Authors should ensure that their data description is fully accurate rather than limited to what they are interested in. Moreover, for these data, as well as all others throughout the study, statements on data reproducibility need to be added, and whenever possible, data should be quantified, and differences between experimental groups analyzed statistically.

5/ Lines 214-227. The data do not suggest a delay of mutant growth plate chondrocytes to enter the hypertrophic stage, since, as described by the authors, the cartilage length between the resting and hypertrophic zones is normal in mutant animals. Moreover, it is incorrect to say that the smaller primary ossification center in mutants is due to a delay in entering hypertrophy and in progressing beyond hypertrophy. If the authors were measuring the length of the POC in mutant elements to that in control elements of equivalent total length (thus one day or two days earlier), they would likely find no problem in POC formation in mutants, but rather a problem in elongating cartilage primordia (likely due to a reduced rate of chondrocyte proliferation).

6/ Lines 237-240. Since the authors failed to detect cell death at the osteochondral junction, it is incorrect to conclude from this negative result that cell death was not impaired in mutant terminal chondrocytes.

© 2024. Published by The Company of Biologists under the terms of the Creative Commons Attribution License (https://creativecommons.org/licenses/by/4.0/).

7/ The data from Foxc mutants generated with Col10a1-Cre is a welcome addition to the revised manuscript. However, the analysis of the mice is insufficient to draw convincing conclusions. At the minimum, it should be shown that Foxc1 and Foxc2 expression is abrogated in Col10a1-Cre mutant animals only in prehypertrophic and hypertrophic chondrocytes, not in precartilaginous condensations and perichondrium cells; and that the onset of chondrocyte hypertrophy is delayed in young fetuses. These data are needed to support the message that Foxc1 and Foxc2 have important cell-autonomous roles in prehypertrophic and hypertrophic chondrocytes. At present, it is hard to explain why Foxc Col10a1-Cre mutant fetuses are much smaller than control littermates, even though hypertrophic chondrocytes are present and look functional, at least at E16.5. Of note, it is puzzling that only two double heterozygous mutants were obtained in seven litters, even though their occurrence (like that of homozygous mutants) should be 1/8.

Text issues
1/ The title of the manuscript refers to functions of Foxc1 and Foxc2 in osteochondral progenitors that are important for progression through chondrocyte hypertrophy and formation of the primary ossification center, but the main novelty of the study proposed by the authors is that the two genes have cell-autonomous functions in growth plate chondrocytes maturing into hypertrophic cells, thus way after the cells have progressed from osteochondral progenitors. The study did not address roles for the two genes in osteochondral progenitors. A change in the title of the manuscript was already recommended at the first review of the manuscript, but this point was not addressed by the authors.

2/ Abstract. The sentence “cartilage formation and mineralization was severely disrupted in the hands and feet” should be changed to “cartilage formation and endochondral ossification were severely disrupted in the paws”. The sentence “later, chondrocyte hypertrophy was slowed, leading to an accumulation of COLX-expressing HCs” needs to be rewritten because the accumulation of Col10a1-expressing cells cannot be due to a delay of chondrocytes to become hypertrophic, but is likely due to a delay in undergoing subsequent cell death or osteoblast differentiation (when a sink gets filled, it is not because the faucet cannot be opened properly, but because the drain is not working properly). Abbreviations should be avoided in the abstract, but if used, should be defined (POC was not defined). Also, the ages of the mice at which data were analyzed needs to be mentioned. Finally, in the abstract and throughout the text, gene acronyms need to be verified for accuracy. For instance, the official acronym for the collagen type X gene is Col10a1, not Colx.

3/ Introduction.
- The first sentence needs to be corrected because the limb bones form through endochondral ossification, but not the limbs themselves, as written.
- Line 40. As written, the origin of the perichondrium cells is not indicated. It should be made clear that these cells derive from lateral plate mesoderm, just like chondrocytes.
- Line 50. Hypertrophic chondrocytes do NOT express MMP9.
- Line 51. Delete the word “remodeling”.
- The text mixes present and future tenses. This needs to be corrected.
- Line 71. What kind of FOXC1 mutations cause Axenfeld Rieger syndrome? Heterozygous or homozygous? Inactivating, overactivating, or other mutations?
- Lines 73 and 74. The word “chromosomal” is awkward. The sentence needs to be rewritten as it is incorrect to say that deletions present with anomalies (patients with deletions present anomalies, but not the deletions themselves). Please specify what types of skeletal anomalies are seen, as this is important with respect to the present study.
- Line 78. The skull is not part of the axial skeleton.
- Line 77-79. It is unclear what the malformations are and whether limbs are affected.
- Line 80. Is the DNA-binding domain the only functional domain of Foxc1 and Foxc2. If not, the conservation of this domain is not sufficient to expect compensation of the loss of one factor by the other.
- Line 90. Specify what “impaired chondrocyte differentiation” is. This is highly relevant to this study,

4/ Results
- Line 105. The sentence is incomplete.

© 2024. Published by The Company of Biologists under the terms of the Creative Commons Attribution License (https://creativecommons.org/licenses/by/4.0/).

- Lines 112-113. The words “share overlapping roles” needs to be replaced “have overlapping roles”.
The words “in parallel” are incorrect to convey the concept proposed by the authors that other factors may share roles with Foxc1 and Foxc2.
- Lines 207-208. The wording that COLX and MMP13 proteins are persistent may not be inaccurate.
While terminally differentiated chondrocytes appear to be delayed in undergoing death or osteoblast differentiation and thus continuing to produce COLX and MMP13 proteins longer than normally, the proteins themselves may not have a longer half-life than in control samples.
- Line 230. The TUNEL assay detects cell death, but it is not specific for cell apoptosis.
- Line 280. “these cells”. Please specify that “these” cells are osteoblast progenitors.

Discussion
- Line 325-328. Sox9-Cre is active in all skeletal progenitors and not solely in chondrocyte progenitors.
Revise wording and conclusions accordingly.
- Line 369. “… more sensitive to the absence of Foxc loss”. Did you mean “… more sensitive to Foxc loss”?
- Line 397. Add “E” before 13.5 and 14.5.

<u>Reviewer 2</u>

Advance summary and potential significance to field

This manuscript provided data in support of an overlapping role for Foxc1 and Foxc2 transcription factors in mouse limb development. Their findings if substantiated provide new insight in the regulation of chondrocyte hypertrophy in endochondral ossification, regulating entry as well as exit of hypertrophy chondrocytes. This is highly relevant to biologist working in skeletal development as well as associated rare diseases of the skeleton.

Comments for the author

The revised manuscript has clarified many of the concerns and included additional data, quantifications, and annotation of figures. Further, to address the critical question of whether the observed changes is a direct effect from expression in hypertrophic chondrocytes or overall delay in the developmental process, the authors provided preliminary data from a conditional inactivation of Foxc1 and Foxc2 using Bac-Col10a1-Cre mouse. This support that the abnormal endochondral ossification is in part intrinsic to the absence or reduced expression level of Foxc1 and Foxc1 in hypertrophic chondrocytes. There are still few minor issues that require further clarification, explanation, and for the discussion to be within the limitations of the data.

1. There is a need to use consistent terminologies such as the gene names for Collagen II and Collagen X are Col2a1 and Col10a1, respectively; while the proteins can be abbreviated to ColII and ColX, or state as COL2 and COL10 etc.

2. It would be important to clarify the nature of the Bac-Col10a1Cre, and the original name should be quoted prior to further abbreviation such as Col10a1-Cre and not ColX-cre, as it is referring to the gene and not protein. Bac-Col10a1Cre is a transgenic mouse, and it does not affect the endogenous Col10a1 expression. While these are preliminary data, the level of Foxc1 and Foxc2 expression should be assessed in these conditional mice, if possible, as the inactivation of Foxc2 with the Prx1-Cre mouse was only 75%.

3. While the use of Col10a1-Cre in part address the cell autonomous contributing by hypertrophic chondrocytes, it does not entirely resolve the issue and that some data can still be interpreted as from a delay in development. The late onset and reduced size of the primary ossification centre (POC) is a developmental problem and illustrate a role in chondrocyte differentiation to hypertrophy in this process. The expanded hypertrophic zone once the growth plate is established in the elongation of bone represents a problem with exiting hypertrophy. Given that apoptosis at the chondro-osseous junction is negligible or not effect, then a possible

© 2024. Published by The Company of Biologists under the terms of the Creative Commons Attribution License (https://creativecommons.org/licenses/by/4.0/).

explanation could be impaired transition to osteoblasts, that also could in part contributes to the mineralization problem at the trabecular region of growth plate.

4. It is odd that the expression of level of Col10a1 mRNA is negligible at E13.5 and authors suggest it is due to the regulation of Col10a1 expression by FOXC1. However, the protein level by immunostaining is readily detectable at E14.5. Are they suggesting there is a sudden change in the transcriptional regulation of Col10a1 within this window of development?

5. The interpretation on the expression of Ihh and downstream targets Ptch1 and Gli1 is rather simplistic and does not seem to be consistent with the data, as there is clear change in the range of IHH signaling in the POC of the Prx1-cre mutant mice. Can author discuss the impact of this expanded range of signalling?

6. Page 18, lines 386-390. The description of “first wave of chondrocyte differentiation occurs independently of HC” is rather unclear. It is a cartilage analgen containing differentiated chondrocytes that are yet to undergo hypertrophy forming the POC. I feel the authors need to clearly define their terminology of chondrogenesis, which is the formation of cartilage from progenitor cells, and hypertrophy as a further differentiation in the process of endochondral ossification.

7. Page 19, lines 412. I am puzzled that the authors claimed the Col10a1-Cre inactivation data did not recapitulate all the endochondral ossification phenotype. Looking at the data, the changes in the growth plate (endochondral ossification) is consistent with the data from Prx1-Cre inactivation.

8. In figure 8, the conclusion that OSX-positive osteoblasts “are not able to migrate into the centre of the newly forming POC, resulting in reduced numbers of OSX-positive osteoblasts and impaired mineralization of the POC” cannot be justified as migration per se was not studied directly.

Reviewer 3

Advance summary and potential significance to field

Previously stated

Comments for the author

The revised manuscript has addressed my concerns, and the inclusion of the ColX-cre cKO mouse is helpful for the interpretations regarding the developmental delay.
I have one minor point that should be addressed for clarity - Osx+ cells were quantified at E16.5 and E17.5, in the anterior and posterior regions of the tibia. At E17.5, it doesn’t seem that the regions quantified are comparable between control and cKO. In general, the replicates in these quantifications and others within the paper (where sections are used to quantify numbers of cells) should be clearly stated in the figure legends, or the methods. I assume they are similar depth sections from more than one mouse, but this is just not stated.
Copy editing suggested for small typographical non-conformities.

First revision

Author response to reviewers' comments

We thank the reviews for their time and thorough evaluation of this manuscript. We appreciate the different perspectives each reviewer provided on our work. These comments have helped reshape our interpretation of our data. We have addressed the issues raised by all reviewers to the best of our abilities and resources. I will address these points below **(Bold text).**

© 2024. Published by The Company of Biologists under the terms of the Creative Commons Attribution License (https://creativecommons.org/licenses/by/4.0/).

Reviewer 1 Comments for the Author:

Almubarak and collaborators have partially improved their study and manuscript in response to the reviewers' recommendations. Several major issues, however, remain to be addressed to convincingly support the new messages of the study. Many minor ones remain to be attended to too.

Scientific issues
1/ The description of the spatial and temporal expression pattern of Foxc1 and Foxc2 in developing appendicular skeletal elements remains suboptimal. Accurately documenting and describing this expression pattern is important to properly interpret the phenotype of embryos lacking both genes. This issue is seen for instance in the lines 143-145. "… Foxc1 and Foxc2 mRNA signals were prominent in the perichondrium and growth plate, including the hypertrophic chondrocytes…". The actual data show that the expression of two genes is prominent in the perichondrium of cartilage primordia (tarsals, metatarsals, and tibial epiphyses); that it progressively declines in the perichondrium as growth plate chondrocytes mature from the proliferative to the hypertrophic stage (tibial metaphyses); and that it is weak in hypertrophic chondrocytes. Given that the expression of the two genes declines as skeletal cells mature, the stronger expression of the genes in distal compared to proximal elements may reflect the fact that distal elements develop later than proximal elements rather than reflect a true patterning difference between proximal and distal limb structures. Reviewers asked for additional data to address more definitively the question of the expression change between distal and proximal structures, but this was not done in the revised manuscript. It is recommended that this point be addressed more convincingly to help explain the apparent proximo-distal defect of mutant limbs, or, at the minimum, that data be accurately described and interpreted. In addition, it is unclear in the data presented in Figure 2 if hypertrophic chondrocytes are positive for Foxc1 and Foxc2 expression at both E14.5 and E16.5, or only at E14.5. It is recommended that the authors show a close-up image at both stages and that they increase the brightness of their images to address this point. It is important to address this second point because a main message that the authors propose for their study is that mutant growth plate chondrocytes are initially delayed in undergoing hypertrophy, but overcome this defect subsequently, and that this delay is due to cell-autonomous roles of Foxc1 and Foxc2 in maturing chondrocytes. Also note that the panels of Figure 2 are not indicated properly in the main text.

We added additional in situ hybridization data in Fig 2 to detect expression of Foxc1 Foxc2 along with Sox9 at E12.5 and E13.5 in the hindlimb. These studies show that Foxc1 and Foxc2 have some overlap with Sox9 expression in the developing limb skeleton. At E12.5 Foxc1 and Sox9 are intensely stained in the digit condensations, while in more proximal regions, Foxc1, Foxc2 and Sox9 are expressed in condensations that vary in intensity amongst each other. Eg the the area with highest Sox9 levels show lower (but detectable) expression of Foxc1 and Foxc2. This pattern holds true for areas of highest Foxc1 or Foxc2 expression with respect to Sox9 levels.

At E13.5 we observed continued overlapping Foxc1 and Sox9 expression in the autopod with Foxc2 expression increasing at this time point. In the tibia anlage we observe intense Sox9 expression in the newly forming chondrocytes with highest levels of Foxc1 and Foxc2 detected in the surrounding presumptive perichondrium, although Foxc1 and Foxc2 can be detected at lower levels in the Sox9-expressing regions.
These findings support our interpretation of the expression data we presented at E14.5 and E16.5 where in less developmentally mature skeletal structures, Foxc1 and Foxc2 expression are prominent in the bone anlage/newly forming chondrocyte and perichondrium. As development proceeds, expression of Foxc1 and Foxc2 decreases in the growth plate chondrocytes and hypertrophic chondrocytes and highest expression levels are restricted to the perichondrium.

© 2024. Published by The Company of Biologists under the terms of the Creative Commons Attribution License (https://creativecommons.org/licenses/by/4.0/).

We also included higher magnification images of the hypertrophic zone to visualize Foxc1 and Foxc2 expression. Additional localization of Foxc1 and Foxc2 in HC is included in Fig 7 where we investigate expression in the Col10a1-cre mouse.

2/ Lines 180-187. As indicated above, a key message of the study proposed by the authors is that the loss of Foxc1 and Foxc2 results in a delay of mutant prehypertrophic chondrocytes to progress to hypertrophy. The expression data for cell differentiation markers, however, do not support this message since Ihh (prehypertrophic marker) has its expression level reduced rather than having its expression domain increased, as it would be if cells were remaining longer than normal at this stage, and since no data are presented to show whether the early stages of cartilage development (before prehypertrophy) proceed in a proper and timely manner and whether the very first hypertrophic chondrocytes form later in mutant compared to control skeletal elements. Of note, the word “delay” (rather than impairment) should be used here and at other places in the study only if evidence is provided that a feature initially occurs later than expected but is resolved at a later time.

We revised the statements in this section to reflect the interpretation of our data presented. Our initial statements were not clear and we agree with the interpretation that overall chondrocyte development was slowed in the Prx1-cre mutants which led to smaller malformed skeletal structures. Our assessment of the formation of a smaller region of COLX-positive cells and delayed chondrocyte hypertrophy was not meant to be taken as the only phenotypic disruption, but rather a notable observation in our mutants. We have changed much of our interpretation to reflect an overall slowing of developmental events.

We have included additional RT-qPCR data from E13.5 embryos (Figure S2) to assess expression of chondrocyte differentiation markers. Although we do appreciate the lack of spatial information these data provides. However these data, along with Figure S1, do indicate that mice lacking Foxc1 and Foxc2 in skeletal progenitors in the limb are able to initiate chondrogenic differentiation events

3/ Lines 188-190. Note that the incomplete recombination of Foxc1 in limbs may reflect the fact that Foxc1 may be expressed in cells that are not targeted by Prx1Cre (such as muscle cells) rather than the proposed explanation that Prx1Cre is not efficiently recombining the gene.

We have added text to lines 209 to reflect this interpretation.

4/ Lines 200-202. It is unexpected that Runx2 expression is not detected in control hypertrophic chondrocytes. It appears to be clearly upregulated in mutant hypertrophic chondrocytes, as described by the authors. However, this upregulation does not seem to be specific for hypertrophic chondrocytes since signals for Runx2 are stronger in several other cells too, including perichondrium/periosteum cells, and proliferating and prehypertrophic chondrocytes. Authors should ensure that their data description is fully accurate rather than limited to what they are interested in. Moreover, for these data, as well as all others throughout the study, statements on data reproducibility need to be added, and whenever possible, data should be quantified, and differences between experimental groups analyzed statistically.

We have added additional information in the figure legends to reflect experimental replicates, phenotypic observational frequencies and statistical analysis of quantitative data. Further information has also been added to the materials and methods. The text reflecting Runx2 localization has been removed, as more detailed analysis would be required to conclude that elevated expression in HC and we wished to use our resources elsewhere.

© 2024. Published by The Company of Biologists under the terms of the Creative Commons Attribution License (https://creativecommons.org/licenses/by/4.0/).

5/ Lines 214-227. The data do not suggest a delay of mutant growth plate chondrocytes to enter the hypertrophic stage, since, as described by the authors, the cartilage length between the resting and hypertrophic zones is normal in mutant animals. Moreover, it is incorrect to say that the smaller primary ossification center in mutants is due to a delay in entering hypertrophy and in progressing beyond hypertrophy. If the authors were measuring the length of the POC in mutant elements to that in control elements of equivalent total length (thus one day or two days earlier), they would likely find no problem in POC formation in mutants, but rather a problem in elongating cartilage primordia (likely due to a reduced rate of chondrocyte proliferation).

We have rewritten this section (Line 236-252) to reflect that the reduced size of hypertrophic chondrocyte zone observed is not specifically a delay in entry into hypertrophy, but rather a result of developmental delay. We also included the reviewer's correct assessment of the length of the POC in the E17.5 mutants vs E16.5 controls.

6/ Lines 237-240. Since the authors failed to detect cell death at the osteochondral junction, it is incorrect to conclude from this negative result that cell death was not impaired in mutant terminal chondrocytes

We have corrected this text (now Lines 262-64) to reflect this comment.

.

7/ The data from Foxc mutants generated with Col10a1-Cre is a welcome addition to the revised manuscript. However, the analysis of the mice is insufficient to draw convincing conclusions. At the minimum, it should be shown that Foxc1 and Foxc2 expression is abrogated in Col10a1-Cre mutant animals only in prehypertrophic and hypertrophic chondrocytes, not in precartilaginous condensations and perichondrium cells; and that the onset of chondrocyte hypertrophy is delayed in young fetuses. These data are needed to support the message that Foxc1 and Foxc2 have important cell-autonomous roles in prehypertrophic and hypertrophic chondrocytes. At present, it is hard to explain why Foxc Col10a1-Cre mutant fetuses are much smaller than control littermates, even though hypertrophic chondrocytes are present and look functional, at least at E16.5. Of note, it is puzzling that only two double heterozygous mutants were obtained in seven litters, even though their occurrence (like that of homozygous mutants) should be 1/8.

We assessed expression of Foxc1 and Foxc2 in the growth plate of E14.5 (when Foxc1 and Foxc2 expression is high in HCs) in control and Col10a-cre mutants. We show a prominent and measurable reduction in Foxc1 and Foxc2 expression in the HC zone of Col10a-cre mutant embryos without affecting expression in the growth plate or perichondrium. We measured expression in the HC by counting fluorescent foci and used a low stringency (3 or more foci/cell; as per the manufacturer's recommendation) to measure expression and capture any expression detected in the mutant HC zone. We also detected COLX protein levels by IF to visualize the HC zone. The length of the COLX-positive regions were measured at E14.5 and E16.5, with only a difference in length detected at E16.5. Furthermore, additional double heterozygous embryos were collected and analyzed to control for the effect of cre expression in Col10a-expressing cells. All of these results have been added to Fig 7.

We did not observe any reduction in ColX protein localization in the E14.5 mutant embryos, indicating entry into hypertrophy was not delayed. This may reflect that loss of Foxc1 or Foxc2 in Col10a-expressing cells has no effect on entry into hypertrophy or timing of deleting Foxc1 or Foxc2 was not early enough. However, the purpose of this experiment was to assess the transition out of chondrocyte hypertrophy with our measured outcomes being the lengths of the COLX-expression zone and the primary ossification centre at E16.5, where a clear separation of the distal and proximal HC zone have been established. Why the mutant embryos are smaller at E16.5 is an interesting question. Smaller bone structures in embryonic development have also been reported in conditional mutants using Col10a-cre mouse lines (Rashid et al 2021 PMID: 34805821; Wang et al 2022: PMID: 35472633). We do observe a smaller POC in the tibia of Col10a-cre mutants with a corresponding reduction in bone length.

© 2024. Published by The Company of Biologists under the terms of the Creative Commons Attribution License (https://creativecommons.org/licenses/by/4.0/).

We would also expect a similar effect in the remainder of the skeleton (including the spine, which is not targeted in Prx1-cre). Due to time limitations, we have not analyzed the entire skeleton, nor have we looked at additional mechanistic explanations of why the POC was smaller in the mutants (ie alterations in HC removal or differentiation into osteoblasts). These are very exciting directions for future research in our laboratories. Lastly we removed the figure of the Col10a-cre whole embryos due to space restrictions.

Text issues

1/ The title of the manuscript refers to functions of Foxc1 and Foxc2 in osteochondral progenitors that are important for progression through chondrocyte hypertrophy and formation of the primary ossification center, but the main novelty of the study proposed by the authors is that the two genes have cell- autonomous functions in growth plate chondrocytes maturing into hypertrophic cells, thus way after the cells have progressed from osteochondral progenitors.
The study did not address roles for the two genes in osteochondral progenitors. A change in the title of the manuscript was already recommended at the first review of the manuscript, but this point was not addressed by the authors.

We have changed the title to take these comments into account

2/ Abstract. The sentence “cartilage formation and mineralization was severely disrupted in the hands and feet” should be changed to “cartilage formation and endochondral ossification were severely disrupted in the paws”. The sentence “later, chondrocyte hypertrophy was slowed, leading to an accumulation of COLX-expressing HCs” needs to be rewritten because the accumulation of Col10a1-expressing cells cannot be due to a delay of chondrocytes to become hypertrophic, but is likely due to a delay in undergoing subsequent cell death or osteoblast differentiation (when a sink gets filled, it is not because the faucet cannot be opened properly, but because the drain is not working properly). Abbreviations should be avoided in the abstract, but if used, should be defined (POC was not defined). Also, the ages of the mice at which data were analyzed needs to be mentioned. Finally, in the abstract and throughout the text, gene acronyms need to be verified for accuracy. For instance, the official acronym for the collagen type X gene is Col10a1, not Colx.

These corrections have been made in the abstract

3/ Introduction.
-The first sentence needs to be corrected because the limb bones form through endochondral ossification, but not the limbs themselves, as written.
Corrected Line 38

-Line 40. As written, the origin of the perichondrium cells is not indicated. It should be made clear that these cells derive from lateral plate mesoderm, just like chondrocytes.
Corrected Line 42

-Line 50. Hypertrophic chondrocytes do NOT express MMP9.
Corrected Line 52

-Line 51. Delete the word “remodeling”.
Corrected Line 53

-The text mixes present and future tenses. This needs to be corrected.
Corrected

-Line 71. What kind of FOXC1 mutations cause Axenfeld Rieger syndrome? Heterozygous or homozygous? Inactivating, overactivating, or other mutations?
We have added text to line 72 to address these queries.

© 2024. Published by The Company of Biologists under the terms of the Creative Commons Attribution License (https://creativecommons.org/licenses/by/4.0/).

Axenfeld Rieger is an autosomal dominant disorder, with haploinsufficiency from inactivating a single *FOXC1* allele being one genetic cause of this disorder. Interestingly patients with chromosomal duplications involving Foxc1 can also present with similar phenotypes as loss of function mutations. However it's not known whether this is a result of increased Foxc1 activity or not. Other lab's attempts to model this feature have been unsuccessful.

-Lines 73 and 74. The word "chromosomal" is awkward. The sentence needs to be rewritten as it is incorrect to say that deletions present with anomalies (patients with deletions present anomalies, but not the deletions themselves). Please specify what types of skeletal anomalies are seen, as this is important with respect to the present study.
Corrected in Line 75-76.

-Line 78. The skull is not part of the axial skeleton.
We changed the wording on line 80, to remove mention of the axial skeleton in this context.

\Line 77-79. It is unclear what the malformations are and whether limbs are affected.
Lines 80 and 81 have been edited to include additional phenotype information.

-Line 80. Is the DNA-binding domain the only functional domain of Foxc1 and Foxc2. If not, the conservation of this domain is not sufficient to expect compensation of the loss of one factor by the other.
Additional information regarding conservation of the transactivation domain has been added to line 84.

-Line 90. Specify what "impaired chondrocyte differentiation" is. This is highly relevant to this study,
We describe the phenotypes in more detail in line 94-98

4/ Results
-Line 105. The sentence is incomplete.
Corrected Line 111

-Lines 112-113. The words "share overlapping roles" needs to be replaced "have overlapping roles". The words "in parallel" are incorrect to convey the concept proposed by the authors that other factors may share roles with Foxc1 and Foxc2.
Corrected on Line 118

-Lines 207-208. The wording that COLX and MMP13 proteins are persistent may not be inaccurate. While terminally differentiated chondrocytes appear to be delayed in undergoing death or osteoblast differentiation and thus continuing to produce COLX and MMP13 proteins longer than normally, the proteins themselves may not have a longer half-life than in control samples.
Corrected Line 232 to reflect this comment.

-Line 230. The TUNEL assay detects cell death, but it is not specific for cell apoptosis.
Corrected line 254

-Line 280. "these cells". Please specify that "these" cells are osteoblast progenitors.
Corrected Line 304

Discussion
-Line 325-328. Sox9-Cre is active in all skeletal progenitors and not solely in chondrocyte progenitors. Revise wording and conclusions accordingly.
Corrected Line 347-49

-Line 369. "… more sensitive to the absence of Foxc loss". Did you mean "… more sensitive to Foxc loss"?
Corrected Line 385

© 2024. Published by The Company of Biologists under the terms of the Creative Commons Attribution License (https://creativecommons.org/licenses/by/4.0/).

-Line 397. Add “E” before 13.5 and 14.5.
Corrected Line 395

***** Reviewer 2 Advance Summary and Potential Significance to Field:
This manuscript provided data in support of an overlapping role for Foxc1 and Foxc2 transcription factors in mouse limb development. Their findings if substantiated provide new insight in the regulation of chondrocyte hypertrophy in endochondral ossification, regulating entry as well as exit of hypertrophy chondrocytes. This is highly relevant to biologist working in skeletal development as well as associated rare diseases of the skeleton.

Reviewer 2 Comments for the Author:
The revised manuscript has clarified many of the concerns and included additional data, quantifications, and annotation of figures. Further, to address the critical question of whether the observed changes is a direct effect from expression in hypertrophic chondrocytes or overall delay in the developmental process, the authors provided preliminary data from a conditional inactivation of Foxc1 and Foxc2 using Bac-Col10a1-Cre mouse. This support that the abnormal endochondral ossification is in part intrinsic to the absence or reduced expression level of Foxc1 and Foxc1 in hypertrophic chondrocytes. There are still few minor issues that require further clarification, explanation, and for the discussion to be within the limitations of the data.

1. There is a need to use consistent terminologies such as the gene names for Collagen II and Collagen X are Col2a1 and Col10a1, respectively; while the proteins can be abbreviated to ColII and ColX, or state as COL2 and COL10 etc.
We have corrected these instances throughout the manuscript

2. It would be important to clarify the nature of the Bac-Col10a1Cre, and the original name should be quoted prior to further abbreviation such as Col10a1-Cre and not ColX-cre, as it is referring to the gene and not protein. Bac-Col10a1Cre is a transgenic mouse, and it does not affect the endogenous Col10a1 expression. While these are preliminary data, the level of Foxc1 and Foxc2 expression should be assessed in these conditional mice, if possible, as the inactivation of Foxc2 with the Prx1-Cre mouse was only 75%.
As described above, we added additional data to Fig 7 to monitor Foxc1 and Foxc2 mRNA expression in the control and Col10a1-cre mutants

3. While the use of Col10a1-Cre in part address the cell autonomous contributing by hypertrophic chondrocytes, it does not entirely resolve the issue and that some data can still be interpreted as from a delay in development. The late onset and reduced size of the primary ossification centre (POC) is a developmental problem and illustrate a role in chondrocyte differentiation to hypertrophy in this process. The expanded hypertrophic zone once the growth plate is established in the elongation of bone represents a problem with exiting hypertrophy. Given that apoptosis at the chondro-osseous junction is negligible or not effect, then a possible explanation could be impaired transition to osteoblasts, that also could in part contributes to the mineralization problem at the trabecular region of growth plate.
We have expanded on the possibilities of how loss of Foxc1 and Foxc2 in the hypertrophic chondrocytes may affect POC formation. Particularly, we discuss the possibility of Foxc1/2 regulating the chondrocyte to osteoblast transition during formation of the POC. These changes have been added to Lines 435-7 of the discussion.

4. It is odd that the expression of level of Col10a1 mRNA is negligible at E13.5 and authors suggest it is due to the regulation of Col10a1 expression by FOXC1. However, the protein level by immunostaining is readily detectable at E14.5. Are they suggesting there is a sudden change in the transcriptional regulation of Col10a1 within this window of development?
In our hands we were unable to detect ColX protein in the hindlimbs of control and prx1-cre mutant embryos at E13.5, however, COLX protein was detected in the forelimb skeletal

© 2024. Published by The Company of Biologists under the terms of the Creative Commons Attribution License (https://creativecommons.org/licenses/by/4.0/).

elements at this age in control embryos but not prx1-cre embryos. We have added this data to Figure S2. Given that forelimbs skeletal structures are at an advanced development state compared to hindlimbs there may be a rapid production of ColX protein in hindlimb structures as hypertrophic chondrocytes form at E14.5. As the qRT-PCR data we present is relative comparison between control and mutant embryos and not an absolute quantification, the expression levels we detect in control embryos may still be at low amounts in the hindlimbs and will increase considerably as more cells enter hypertrophy

5. The interpretation on the expression of Ihh and downstream targets Ptch1 and Gli1 is rather simplistic and does not seem to be consistent with the data, as there is clear change in the range of IHH signaling in the POC of the Prx1-cre mutant mice. Can author discuss the impact of this expanded range of signalling?
This is an interesting possibility we initially did not consider. Our interpretation of these data is the IHH-PTHLH signal is not sufficiently partitioned and abundant PTHLH and PTHLHr expression is present in the HC and POC. Given that PTHLH has inhibitory effects on chondrocyte hypertrophy, the expanded range of activity may contribute to the reduced size of the HC zone in the prx1-cre mutants. We have added this explanation to the results and discussion sections (Lines 202-05 and Lines 400-08, respectively)

6. Page 18, lines 386-390. The description of “first wave of chondrocyte differentiation occurs independently of HC” is rather unclear. It is a cartilage analgen containing differentiated chondrocytes that are yet to undergo hypertrophy forming the POC. I feel the authors need to clearly define their terminology of chondrogenesis, which is the formation of cartilage from progenitor cells, and hypertrophy as a further differentiation in the process of endochondral ossification.
We have clarified our statements in this section using the suggested terminology.

7. Page 19, lines 412. I am puzzled that the authors claimed the Col10a1-Cre inactivation data did not recapitulate all the endochondral ossification phenotype. Looking at the data, the changes in the growth plate (endochondral ossification) is consistent with the data from Prx1-Cre inactivation.
The original statement was referring to all the phenotypes observed in Prx1-cre mouse rather than the expanded HC and truncated POC at E16.5 on the Col10a-cre mutants

8. In figure 8, the conclusion that OSX-positive osteoblasts “are not able to migrate into the centre of the newly forming POC, resulting in reduced numbers of OSX-positive osteoblasts and impaired mineralization of the POC” cannot be justified as migration per se was not studied directly.
We have corrected the text and instead use the terminology “populate the POC”

***** Reviewer 3 Advance Summary and Potential Significance to Field:

Previously stated

Reviewer 3 Comments for the Author:
The revised manuscript has addressed my concerns, and the inclusion of the ColX-cre cKO mouse is helpful for the interpretations regarding the developmental delay.

I have one minor point that should be addressed for clarity -

Osx+ cells were quantified at E16.5 and E17.5, in the anterior and posterior regions of the tibia. At E17.5, it doesn’t seem that the regions quantified are comparable between control and cKO.

In quantifying our microscopy data, our goals were to numerically represent the observations we saw between the controls and mutants. For figure 8 we wanted to assess the noticeable asymmetry in OSX density observed in the mutants, and therefore evaluated the entire primary ossification center as to reduce any bias. Given the differences in sizes of POC we understand that the sampling area was different.

© 2024. Published by The Company of Biologists under the terms of the Creative Commons Attribution License (https://creativecommons.org/licenses/by/4.0/).

However, as we were normalizing these counts to total cell number (DAPI+ nuclei), we felt these sample size differences would not affect our interpretation of the results.

In general, the replicates in these quantifications and others within the paper (where sections are used to quantify numbers of cells) should be clearly stated in the figure legends, or the methods. I assume they are similar depth sections from more than one mouse, but this is just not stated.
We have added additional statements to the methods sections and figure legends to clarify this information.

Copy editing suggested for small typographical non-conformities.

Second decision letter

MS ID#: DEVELOP/2024/202798

MS TITLE: Foxc1 and Foxc2 function in growth plate chondrocyte maturation towards hypertrophy and regulate formation of the primary ossification center in mouse embryonic limb skeleton development.

AUTHORS: Asra Almubarak, Qiuwan Zhang, Cheng-Hai Zhang, Noor Abdelwahab, Tsutomu Kume, Andrew B Lassar, and Fred B Berry
ARTICLE TYPE: Research Article

I am happy to tell you that your manuscript has been accepted for publication in Development, pending our standard publication integrity checks.

Reviewer 1

Advance summary and potential significance to field

This paper expands current knowledge of the functions of the transcription factors Foxc1 and Foxc2 in embryonic skeletogenesis. It thus expands understanding of the regulatory networks that govern this process and will be helpful for many more studies.

Comments for the author

The authors greatly improved their study and manuscript according to the comments of all reviewers. The paper is, in my opinion, ready for publication.

Reviewer 2

Advance summary and potential significance to field

This manuscript provided data in support of an overlapping role for Foxc1 and Foxc2 transcription factors in mouse limb development. Their findings provided new insight in the regulation of chondrocyte hypertrophy in endochondral ossification, regulating entry as well as exit of hypertrophy chondrocytes. This is highly relevant to biologist working in skeletal development as well as associated rare diseases of the skeleton.

Comments for the author

The authors have satisfactorily addressed my concerns. I have no further comments.

© 2024. Published by The Company of Biologists under the terms of the Creative Commons Attribution License (https://creativecommons.org/licenses/by/4.0/).
